# Transcriptome-wide root causal inference

**DOI:** 10.1371/journal.pcbi.1013461

**Published:** 2025-09-02

**Authors:** Eric V. Strobl, Eric R. Gamazon

**Affiliations:** 1 Department of Biomedical Informatics, University of Pittsburgh, Pittsburgh, Pennsylvania, United States of America; 2 Department of Medicine, Vanderbilt University Medical Center, Nashville, Tennessee, United States of America; Tufts University, UNITED STATES OF AMERICA

## Abstract

Root causal genes correspond to the first gene expression levels perturbed during pathogenesis by genetic or non-genetic factors. Targeting root causal genes has the potential to alleviate disease entirely by eliminating pathology near its onset. No existing algorithm has been designed to discover root causal genes from observational data alone. We therefore propose the Transcriptome-Wide Root Causal Inference (TWRCI) algorithm that identifies root causal genes and their causal graph using a combination of genetic variant and unperturbed bulk RNA sequencing data. TWRCI uses a novel competitive regression procedure to annotate cis and trans-genetic variants to the gene expression levels they directly cause. The algorithm simultaneously determines the sequence in which gene expression changes propagate through the system to pinpoint the underlying causal graph and estimate root causal effects. TWRCI outperforms alternative approaches across a diverse group of metrics by directly targeting root causal genes while accounting for distal relations, linkage disequilibrium, patient heterogeneity and widespread pleiotropy. We demonstrate the algorithm by uncovering the root causal mechanisms of two complex diseases, which we confirm by replication using independent genome-wide summary statistics.

## Author summary

Many diseases progress through causal chains. The earliest step detectable in gene expression is a small set of *root causal genes*: expression levels that change first after genetic or non-genetic triggers. Because gene expression is relatively easy to perturb, focusing on these early changes offers a tractable route to stopping disease with a sparse set of interventions. Yet most existing tools either require expensive perturbation screens or fail to distinguish true early causes from downstream consequences. Transcriptome-Wide Root Causal Inference (TWRCI) uses widely available genotype data and bulk RNA-seq to identify these first expression events and quantify their patient-specific effects. TWRCI assigns each genetic variant to the single target it most directly influences—either a gene or the disease outcome—via a head-to-head prediction test, reconstructs the causal chain among genes, and estimates each gene’s patient-specific *root causal effect*, integrating genetic and non-genetic drivers into an interpretable effect size. In simulations and two diseases, TWRCI outperformed alternatives, recovered compact sets of early-acting genes consistent with known biology, detected variants that act directly on disease outside expression, and replicated across cohorts. Most variation in root causal effects was non-genetic, pointing to environmental triggers.

## Introduction

Genetic and non-genetic factors can modulate gene expression levels, ultimately contributing to the development of disease. Root causal gene expression levels—or *root causal genes* for short—correspond to the genes whose *initial* changes in expression trigger a pathogenic cascade ultimately leading to a disease and subsequent phenotypic outcomes [1]. Root causal genes initiate pathogenesis, unlike *core genes* that directly cause the disease phenotype and thus reside at the end of the pathogenic pathway [2]. Root causal genes also generalize *driver genes* that primarily account for the effects of somatic mutations in protein-coding sequences in cancer [20]. Additionally, root causal genes differ from *master regulators*, which control many downstream genes but do not necessarily drive disease [4].

Identifying root causal genes is pivotal for pinpointing drug targets that intervene early in pathogenesis, potentially halting downstream disease progression [5]. The task is complicated by complex diseases, where the causal effects of root causal genes may vary across patients, even within the same diagnostic category. Our recently proposed *omnigenic root causal model* posits that a small number of root causal genes exert strong causal effects on a patient’s diagnosis while influencing the expression of nearly all downstream genes (hence, “omnigenic”) [1]. The widespread influence creates extensive gene-diagnosis correlations, giving the appearance of a “complex” disease. Consequently, we aim to estimate the personalized causal effects of root causal genes in each patient, enabling the identification of those genes with significant influence in driving disease development.

Only one existing algorithm accurately estimates the personalized causal effects of root causal genes [1], but it relies on genome-wide Perturb-seq, or high-throughput perturbations with single-cell RNA sequencing readouts [6,7]. Perturb-seq is currently costly and challenging to implement across diverse cell types. To address this, we propose a method to infer personalized causal effects of root causal genes using widely available observational (non-experimental) datasets, such as bulk RNA sequencing and genetic variant data. The task is complex because observational datasets lack experimental controls, requiring robust strategies to account for confounding factors without relying on perturbation-based causal inference.

We make the following contributions in this paper:

1. We introduce the conditional root causal effect (CRCE), a metric quantifying the causal impact of genetic and non-genetic factors directly affecting a gene expression level on the disease phenotype.
2. We propose a novel strategy called *Competitive Regression* that accurately annotates cis- and trans-acting genetic variants to the gene expression levels or phenotype they directly influence, without relying on conservative statistical significance thresholds.
3. We create an algorithm called *Transcriptome-Wide Root Causal Inference* (TWRCI) that leverages the annotations to construct a personalized causal graph summarizing the CRCEs of gene expression levels. The algorithm only relies on genetic variant and bulk RNA sequencing observational data.
4. We show with confirmatory replication that TWRCI identifies only a few root causal genes with large personalized causal effects in each patient—even in complex diseases—consistent with the omnigenic root causal model. Moreover, *non-genetic* root causes account for the majority of the variance in CRCEs, in contrast to the genetic-centric focus of driver genes [8] and other causal (but not *root* causal) inference methods, such as the Transcriptome-Wide Association Study [9] and Mendelian Randomization [10].

**Fig 1:**
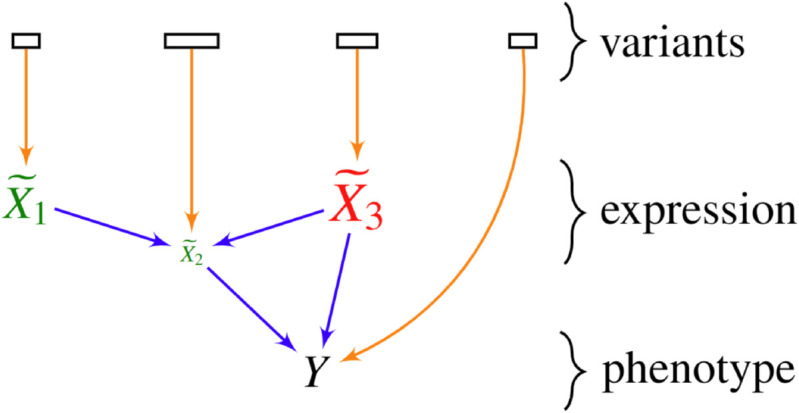
Toy example of a personalized root causal mechanism inferred by TWRCI for an individual patient. Rectangles represent sets of genetic variants, which may be in linkage disequilibrium within or across sets. Each set directly influences either a gene expression level in $\tilde{X}$ or the phenotype *Y*. Vertex size indicates the magnitude of the CRCE, with larger vertices reflecting greater root causal impact. Vertex color denotes CRCE direction: green for positive (phenotype-promoting) and red for negative (phenotype-preventing) effects. TWRCI performs three integrated tasks: variant annotation (orange), causal graph reconstruction (blue) and CRCE estimation (green/red).

We provide an example of the output of TWRCI in Fig 1. TWRCI first uses widely available genome-wide association study (GWAS) summary statistics to select variants associated with a phenotype and then uses more fine-grained individual-level data to annotate the selected cis- and trans-acting genetic variants to the expression level or phenotype they *directly* regulate. We prove that the direct causal annotations allow the algorithm to uniquely reconstruct the causal graph between the gene expression levels that cause the phenotype as well as estimate their CRCEs. The algorithm summarizes the CRCEs in the graph by weighting and color-coding each vertex, where vertex size reflects effect magnitude, green indicates phenotype-promoting effects, and red denotes phenotype-preventing effects. TWRCI thus provides a succinct, patient-specific summary of root causal genes and their root causal effect sizes using observational data alone. TWRCI outperforms combinations of existing algorithms across all subtasks: annotation, graph reconstruction, and CRCE estimation. No existing algorithm performs all subtasks simultaneously.

## Results

### Overview of TWRCI

#### Setup.

**Fig 2:**
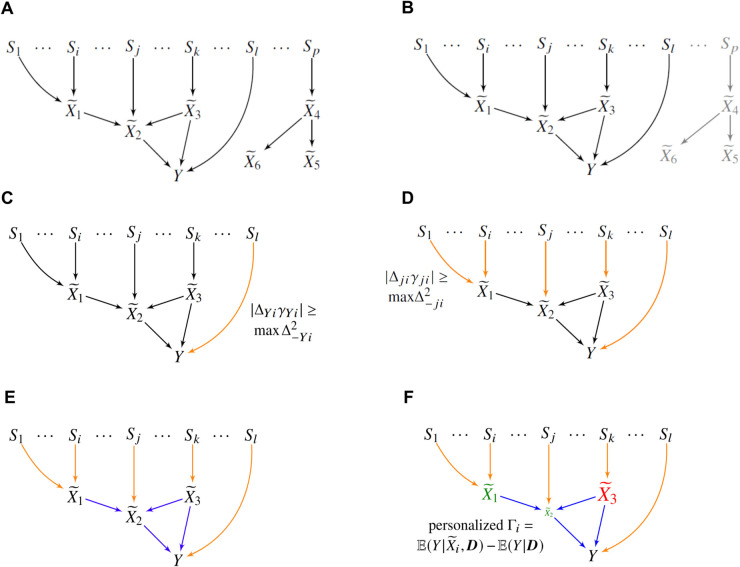
Overview of the TWRCI algorithm. (A) A detailed representation of the personalized causal graph from Fig 1 illustrating the true causal relationships (unobserved in practice) among genetic variants S, gene expression levels $\tilde{X}$, and the disease phenotype *Y*. (B) TWRCI begins with variable selection, retaining only variants and gene expression levels correlated with *Y*, along with their common confounding factors, highlighted in black. (C) The algorithm then uses Competitive Regression to find the variants that directly cause *Y* in orange. (D) Competitive Regression is iteratively applied to annotate variants directly influencing each gene expression level, also marked in orange. (E) TWRCI then employs causal discovery to infer direct causal relationships between gene expression levels and *Y*, depicted in blue. (F) Finally, TWRCI assigns weights to each gene expression vertex ${\tilde{X}}_{i}\in \tilde{R}$ based on the magnitude of its CRCE $\Gamma_{i}$, with colors indicating effect direction: green for phenotype-promoting (positive) and red for phenotype-preventing (negative). Thus, TWRCI reconstructs a patient-specific causal graph, as exemplified in Fig 1.

We aim to estimate the personalized causal effects of root causal genes, which initiate disease pathogenesis, rather than general causal effects. To achieve this, we define a generative causal process involving a set of genetic variants S, the transcriptome $\tilde{X}$, and the disease phenotype *Y*. This process is represented as a directed graph, as shown in Fig 2A, where the variants cause the transcriptome, and the transcriptome in turn causes the phenotype. Directed edges denote direct causal relations between variables. In practice, S comprises millions of genetic variants, and $\tilde{X}$ includes thousands of gene expression levels. As detailed in Methods *Causal Modeling*, RNA sequencing does not directly measure $\tilde{X}$ but instead yields observed expression values X, which are affected by Poisson-distributed measurement noise and batch effects.

#### Variable selection.

Simultaneously handling millions of variants and thousands of gene expression levels currently requires expensive computational resources. Moreover, most variants and gene expression levels do not inform the discovery of root causal genes for a particular phenotype *Y*. TWRCI thus first performs variable selection by eliminating variants and gene expression levels unnecessary for root causal inference (Fig 2B).

TWRCI begins by employing GWAS summary statistics solely as an initial filtering step to reduce the set of genetic variants S to a computationally manageable subset T. We apply a deliberately liberal significance threshold (e.g., $\alpha =5\;\times \;10^{-5}$) during this stage, prioritizing sensitivity to ensure that all potentially causal variants are retained—even if this admits many false positives. This liberal threshold captures a broad range of variants, including those with only weak marginal associations and those in regions of high linkage disequilibrium (LD).

Crucially, we do not make use of an external LD reference panel. After the initial filtering step, we conduct all subsequent analyses using individual-level genotype, gene expression, and phenotype data. This design allows us to estimate LD and covariance structures directly from the study sample, avoiding potential mismatches with external panels. While this requirement limits applicability to settings where individual-level data are available—unlike standard Mendelian Randomization or Transcriptome-Wide Association Study methods that can operate on GWAS summary statistics alone—it enables more robust inference by leveraging the full richness of the sample and reduces the risk of bias from reference panel discrepancies. In summary, we use summary statistics only for computationally tractable variable selection, while all substantive inference relies on individual-level data.

The algorithm then uses the individual-level data to identify the subset of gene expression levels $\tilde{R}\subseteq \tilde{X}$ that it can predict better than chance using T. We prove that $T\;\cup \;\tilde{R}$ retains all of the variants and gene expression levels that cause *Y* in Methods *Variable Selection*. We also refer the reader to the same section for details on the discovery of additional nuisance variables required to address confounding.

#### Annotation by competitive regression.

TWRCI next annotates both cis and trans-acting variants to the gene expression level that they directly cause in $\tilde{R}$ (Fig 2D). The algorithm also annotates variants to the phenotype *Y* in order to account for horizontal pleiotropy, where variants bypass $\tilde{R}$ and directly cause *Y* (Fig 2C). TWRCI achieves both of these feats through a novel process called *Competitive Regression*. Importantly, TWRCI does not assume the existence of horizontal pleiotropy, but rather *allows* for its possibility. Genetic variants may influence phenotypes through mechanisms independent of measured gene expression, such as alternative splicing, RNA stability, or temporal regulation of expression. Accounting for these alternative pathways is critical, as failure to do so can introduce confounding bias. Indeed, a growing body of evidence suggests that horizontal pleiotropy is widespread in human genetics [11,12].

We provide a comprehensive description of Competitive Regression in Methods *Annotation for Horizontal Pleiotropy* and *Annotation and Causal Order*, but provide the intuition here. Competitive Regression first evaluates two conditions to annotate a variant to the phenotype *Y*: (1) whether the variant $T_{i}\in T$ predicts *Y* when considering other variants, (2) whether the same variant *T*_*i*_ still predicts *Y* when considering other variants *and* gene expression levels $\tilde{R}$. The key distinction is that the first step identifies all associations—including both direct effects and those mediated by gene expression—while the second step explicitly blocks all indirect (mediated) pathways by including $\tilde{R}$ in the conditioning set. We infer a direct, unmediated effect on the phenotype only if *T*_*i*_ remains predictive of *Y* after controlling for gene expression. This separation ensures that Competitive Regression can distinguish true direct effects from those acting via gene expression mediation.

In practice, predictions are imperfect in finite samples, so Competitive Regression uses a “competitive” strategy to ensure that decisions are robust to statistical noise. The procedure specifically compares the predictive strength of a variant *T*_*i*_ on *Y* (from conditions (1) and (2) above) against its predictive strength on all the gene expression levels $\tilde{R}$. If the predictive strength of *T*_*i*_ on *Y* is stronger than its predictive strength on $\tilde{R}$, so that *Y* “beats” $\tilde{R}$ for *T*_*i*_, then TWRCI annotates *T*_*i*_ to *Y* (Fig 2C). Notice that this approach accounts for horizontal pleiotropy—where a variant directly affects *Y* (i.e., $T_{i}\to Y$)—by conditioning on $\tilde{R}$, which blocks indirect pathways such as $T_{i}\to \tilde{R}\to Y$. In contrast, fine-mapping methods do not distinguish direct from indirect effects [13]. Competitive Regression also forces *Y* to compete against $\tilde{R}$ in order to avoid statistical thresholds like p-values, posterior inclusion probabilities, or hyperparameters, which can miss subtle but important effects. We rigorously prove the correctness of the annotation procedure to *Y* in Methods *Annotation for Horizontal Pleiotropy*.

TWRCI next removes *Y* from consideration and applies similar logic to the gene expression levels $\tilde{R}$. The algorithm annotates a variant *T*_*j*_ to ${\tilde{R}}_{k}\in \tilde{R}$ by first assessing the predictive strength of *T*_*j*_ on ${\tilde{R}}_{k}$ while considering other variants, and then other variants and other expression levels. TWRCI ensures that such predictive strengths of *T*_*j*_ on ${\tilde{R}}_{k}$ outweigh the variant’s predictive strengths on the other expression levels to confirm a direct causal link between *T*_*j*_ and ${\tilde{R}}_{k}$. This process guarantees that cis- and trans-acting variants are accurately annotated to the gene expression levels they directly regulate, thereby excluding indirect relationships, such as trans effects mediated through cis mechanisms. TWRCI next eliminates ${\tilde{R}}_{k}$ from consideration if ${\tilde{R}}_{k}$ is independent of all variants *not* annotated to it (conditional on the remaining gene expression levels and the variants annotated to ${\tilde{R}}_{k}$), ensuring all relevant causal relationships are captured. The algorithm finally iterates this process, selecting and annotating variants to subsequent expression levels until all gene expression levels in $\tilde{R}$ and the phenotype *Y* have been addressed.

Note that, due to finite sample size and statistical noise, Competitive Regression may occasionally annotate variants to gene expression levels or the phenotype that do not directly cause those outcomes (i.e., false positives). However, the method is robust to such errors in practice. As sample sizes increase to infinity, any variant lacking a direct causal effect on a gene expression level or the phenotype will be assigned to some gene expression level or the phenotype, and its corresponding (debiased) regression coefficient will converge to zero. Thus, while statistical false positives may be observed in finite samples, they can be distinguished from true direct causal variants by examining the magnitudes of the regression coefficients: only those with substantial effect sizes are interpreted as genuine discoveries, while those with near-zero coefficients reflect the lack of a direct causal relationship. This property preserves the interpretability and reliability of the annotations produced by Competitive Regression.

The reliability of Competitive Regression hinges on three standard assumptions used in instrumental variable analysis: no reverse causation, relevance, and exchangeability [14]. The *no reverse causation* assumption states that *Y* is a variable with no downstream effects, so it cannot cause the variants or gene expression levels. The assumption is often justified because, in attempting to discover root causal genes, *Y* typically denotes a fixed chronic diagnostic label (not to be confused with the disease itself). In our approach, we use tissue-specific gene expression measured in the disease-relevant tissue, where forward causality from expression to diagnosis is more plausible, because multiple orthogonal lines of evidence indicate that complex-trait effects concentrate in trait-matched tissues. For example, genome-wide partitioning of heritability shows enrichment near genes specifically expressed in relevant tissues [15]. Moreover, large-scale regulatory maps report widespread GWAS–cis-QTL colocalization in biologically expected organs and cell states (e.g., liver for lipid traits) [16–18]. These observations increase the prior probability of forward causality when coupled with prior physiological knowledge that disease-relevant tissues house causal pathways directed towards the phenotype. Note that the no reverse causation assumption does not require that gene expression changes always precede the assignment of the diagnosis; such changes can occur before or after the diagnosis is established. However, we expect that downstream effects triggered by the diagnosis, such as medications or behavioral change, are generally limited in their impact on gene expression within the relevant tissue, except for specific targeted pathways (e.g., inflammation or lipid metabolism) [19]. By focusing on disease-relevant tissues, our framework minimizes the risk that observed associations are driven by downstream or systemic changes rather than root causal processes.

Next, *relevance* means that at least one variant in ***T*** directly causes each gene expression level in $\tilde{R}$. The assumption usually holds because ***T*** contains millions of variants far outnumbering the thousands of gene expression levels in $\tilde{R}$. Relevance is also empirically supported by large-scale eQTL studies such as GTEx, which have identified significant cis-eQTLs for the majority of expressed genes across most tissues [16]. Moreover, the assumption is expected to weaken over time as advancements in deep sequencing facilitate the identification of increasingly rare variants [20].

On the other hand, *exchangeability* assumes that T and other sets of direct causal variants not in T share no latent confounders; this assumption holds approximately due to the weak causal relations emanating from variants to gene expression and the phenotype. We adjust for the first few principal components to further minimize potential confounding from population structure. Exchangeability also weakens as T grows larger.

Overall, Competitive Regression offers several advantages for genomics research. First, it captures both cis and trans effects, unlike methods that focus only on nearby variants, enabling a more complete understanding of gene regulation in diseases. Second, it accounts for horizontal pleiotropy, which is critical for distinguishing variants that bypass gene expression to directly influence disease risk. Third, it eliminates the need for arbitrary statistical thresholds, such as p-values or posterior inclusion probabilities, by automatically constructing data-driven thresholds based on the relative predictive strength of variants. The data-driven thresholds ultimately enhance sensitivity and robustness as empirically shown in our experiments.

#### Causal discovery and CRCE estimation.

Annotation only elucidates the direct causal relations from variants to gene expression, but it does not recover the causal relations between gene expression or the causal relations from gene expression to the phenotype. We want TWRCI to recover the *entire* biological mechanism from variants all the way to the phenotype.

Let K represent the sequence of variables as they are removed by TWRCI during analysis, beginning with *Y* and proceeding stepwise through the gene expression levels. In this scheme, *Y* is the first variable removed and becomes the last entry in K, the next variable removed becomes second-to-last in K, and so on, so that the variable removed last appears first in K. TWRCI then runs a causal discovery algorithm with K to uniquely identify the causal graph over $\tilde{R}\;\cup \;Y$ (Fig 2E). The algorithm also estimates the personalized or *conditional root causal effect* (CRCE) of each gene expression level that causes *Y*:

$$\Gamma_{i}=𝔼(Y|\overset{non-genetic}{\overbrace{E_{i}}}\cup \overset{genetic}{\overbrace{S_{i}}},D)-𝔼(Y|D),\;=𝔼(Y|\tilde{X_{i}},D)-𝔼(Y|D),$$ (1)

where we choose $D\subseteq \tilde{R}\;\cup \;T$ carefully to ensure that the second equality holds (Methods *Conditional Root Causal Effects*). The CRCE $\Gamma_{i}$ of ${\tilde{X}}_{i}\in \tilde{R}$ thus measures the causal effect of the genetic factors $S_{i}$ and the non-genetic factors *E*_*i*_ on *Y* that perturb ${\tilde{X}}_{i}$ first. The CRCE values differ between patients, so TWRCI can recover different causal graphs by weighing each vertex according to the patient-specific CRCE values Γ=γ (Fig 2F). The gene ${\tilde{X}}_{i}$ is a *personalized root causal gene* if $|\gamma_{i}|>0$. The *omnigenic root causal model* posits that |γ|≫0 for only a small subset of genes in each patient even in complex disease.

### TWRCI accurately annotates, reconstructs and estimates in silico

No existing algorithm recovers CRCEs from observational data alone. However, existing algorithms can annotate variants using different criteria and reconstruct causal graphs from observational data. We therefore compared TWRCI against state-of-the-art algorithms in annotation and causal graph reconstruction using 100 semi-synthetic datasets, incorporating real variant data with LD and simulated gene expression and phenotype data that are interdependent according to random causal graphs (Methods *Semi-Synthetic Data*).

Many different annotation methods exist with different objectives. Most methods, nevertheless, annotate variants by at least considering proximity to the transcription start site (TSS), with the hope that variants near the TSS of a gene will *directly* affect that gene’s expression level; for example, a variant in the exonic region of a gene may compromise its mRNA stability, while a variant in the promoter region may affect its transcription rate. We thus compare a diverse range of methods in *direct causal* annotation, or assigning variants to the gene expression levels they directly cause. This criterion accommodates other annotation objectives from a mathematical perspective as well—solving direct causation automatically solves causation (fine-mapping), colocalization, and correlation as progressively more relaxed cases. Further, we are interested in resolving direct causal variants even within loci in high LD similar to fine-mapping but unlike clumping or pruning techniques. We thus compare nearest TSS, a one mega-base cis-window (If multiple genes were present in the window, then we assigned the variant to the gene with the nearest TSS.), the causal transcriptome-wide association study (cTWAS) [21], the maximally correlated gene within the cis-window (cis-eQTL) [22], colocalization with approximate Bayes factors (ABF) [23], and colocalization with Sum of Single Effects model (SuSiE) [24] without performing clumping or pruning. We then performed causal graph reconstruction using SIGNET [25,26], RCI [27], GRCI [28] and the PC algorithm [29,30]. We evaluated TWRCI against all combinations of annotation and graph reconstruction methods. See Methods *Comparators* and *Metrics* for a detailed description of comparator algorithms and evaluation metrics, respectively. To ensure a fair comparison, we evaluated all methods using an identical pre-filtered set of variants and gene expression levels, as feature selection was necessary to ensure scalability for all data-driven inference algorithms. Consequently, differences in empirical results reflect true differences in methodological performance rather than variations in the number or quality of input features. All statements about empirical results mentioned below hold at a Bonferroni corrected threshold of 0.05 divided by the number of comparator algorithms according to two-sided paired t-tests.

**Fig 3:**
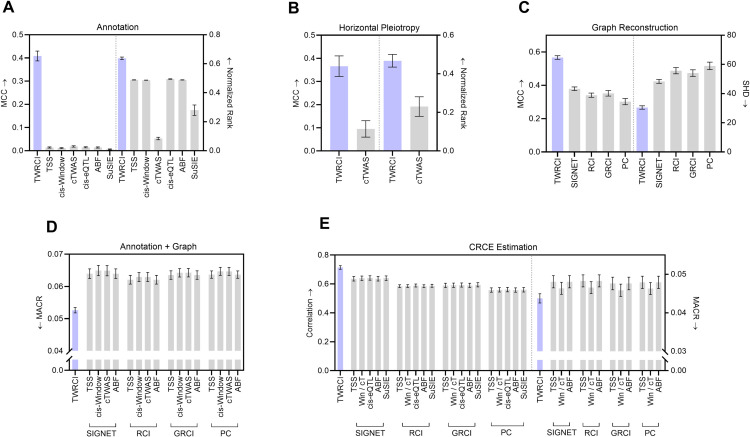
Semi-synthetic data results in terms of (A) direct causal annotation, (B) annotation focused on horizontal pleiotropy only, (C) graph reconstruction, (D) combined annotation and graph reconstruction, and (E) CRCE estimation accuracy. Four of the graphs summarize two evaluation metrics. Arrows near the y-axis denote whether a higher (upward arrow) or a lower (downward arrow) score is better. We do not plot the results of cis-eQTL and SuSiE in (D) and (E) when they exhibit much worse performance. The cis-window and cTWAS algorithms have the exact same CRCE estimates in (E) because accounting for horizontal pleiotropy in cTWAS does not change the conditioning set D in Eq (1); we thus denote cis-Window and cTWAS as Win/cT for short. TWRCI in purple outperformed all algorithms across all nine evaluation metrics. Error bars correspond to 95% confidence intervals.

We first summarize the accuracy results for annotation of direct causes only. All existing annotation algorithms utilize heuristics such as location, correlation or colocalization to infer causality. Only TWRCI provably identifies the direct causes of each gene expression level (Theorem 1 in Methods *Conditional Root Causal Effect Estimation*). Empirical results corroborate this theoretical conclusion. TWRCI achieved the highest accuracy as assessed by Matthew’s correlation coefficient (MCC) to the true direct causal variants of each gene expression level and phenotype (Fig 3A left); we break down MCC into precision and recall in Fig A panel a in S1, where TWRCI also performed in the best in both cases. The algorithm further ranked the ground truth direct causal variants the highest by assigning the ground truth causal variants larger regression coefficient magnitudes than non-causal variants (Fig 3A right). Both TWRCI and cTWAS account for horizontal pleiotropy, but TWRCI again outperformed cTWAS even when we only compared the true and inferred variants that directly cause the phenotype using MCC and the normalized rank (Fig 3B), as well as precision and recall (Fig A panel b in S1). We conclude that TWRCI annotated the genetic variants to their direct effects most accurately.

We obtained similar results with causal graph reconstruction. TWRCI obtained the highest MCC and the lowest structural hamming distance (SHD) to the ground truth causal graphs (Fig 3C). Furthermore, TWRCI achieved the highest precision and recall (Fig A panel c in S1). We then assessed the performance of combined annotation and graph reconstruction using the mean absolute correlation of the residuals (MACR), or the mean absolute correlation between the *indirect* causes of a gene expression level and the residual gene expression level obtained after partialing out the inferred *direct* causes; if an algorithm annotates and reconstructs accurately, then each gene expression level should not correlate with its indirect causes after partialing out its direct causes, so the MACR should attain a small value. TWRCI accordingly achieved the lowest MACR as compared to all possible combinations of existing algorithms (Fig 3D). The cis-eQTL and SuSiE algorithms obtained MACR values greater than 0.3 because many cis-variants did not correlate or colocalize with the expression level of the gene with the nearest TSS; we thus do not plot the results of these algorithms. We conclude that TWRCI used annotations to reconstruct the causal graph most accurately by provably accounting for both cis and trans-acting variants.

We finally analyzed CRCE estimation accuracy. Computing the CRCE requires access to the inferred annotations and causal graph. We therefore again evaluated TWRCI against all possible combinations of existing algorithms. The CRCE estimates of TWRCI attained the largest correlation to the ground truth CRCE values (Fig 3E left). Further, if an algorithm accurately estimates the components $𝔼(Y|{\tilde{X}}_{i},D)$ and 𝔼(Y|D) of the CRCE in Eq (1), then the residual $Y\;-\;𝔼(Y|{\tilde{X}}_{i},D)$ should not correlate with $S_{i}\;\cap \;T$. TWRCI accordingly obtained the lowest mean absolute correlation of these residuals (MACR) against all combinations of algorithms (Fig 3E right). The cis-eQTL and SuSiE algorithms again attained much worse MACR values above 0.4 because they failed to annotate many causal variants to their gene expression levels. We conclude that TWRCI outperformed existing methods in CRCE estimation. TWRCI therefore annotated, reconstructed and estimated the most accurately according to all eleven evaluation criteria. The algorithm also completed within about 3 minutes for each dataset (Fig B in S1).

### Chronic and exaggerated immunity in COPD

We next ran the algorithms using summary statistics of a large GWAS of COPD [32] consisting of 13,530 cases and 454,945 controls of European ancestry. We downloaded individual variant-expression-phenotype data of lung tissue from GTEx [16] with 96 cases and 415 controls. We also replicated results using an independent GWAS consisting of 4,017 cases and 162,653 controls of East Asian ancestry [32]. We mitigated the effects of population stratification, sequencing platform, sequencing protocol, biological sex and age by quality control (Methods *Quality Control*). COPD is a chronic inflammatory condition of the airways or the alveoli that leads to persistent airflow obstruction [33]. Exposure to respiratory infections or environmental pollutants can also trigger acute on chronic inflammation called COPD exacerbations that worsen the obstruction.

#### Accuracy.

**Fig 4:**
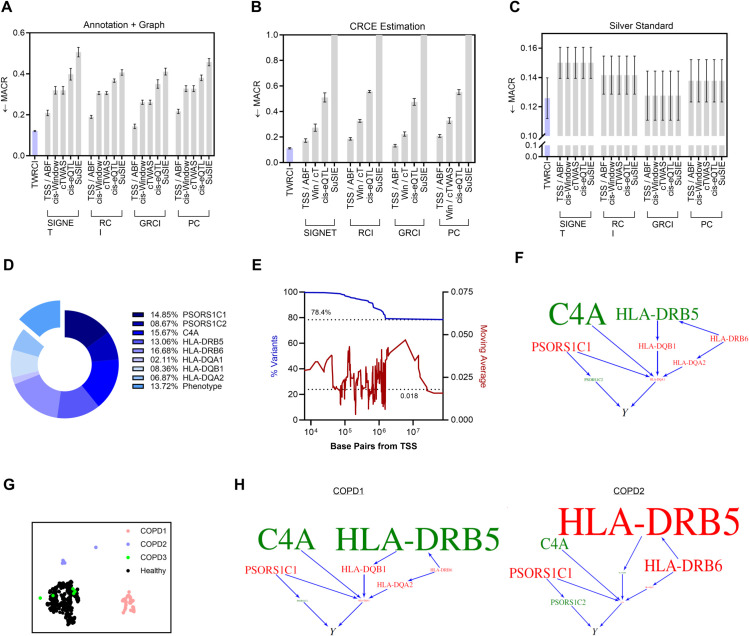
Results for COPD. (A) TWRCI outperformed all other combinations of algorithms in direct causal annotation and graph reconstruction by achieving the lowest MACR; error bars correspond to one standard error of the mean in accordance with the one standard error rule of cross-validation [31]. (B) TWRCI similarly achieved the lowest MACR for CRCE estimation. (C) Silver standard genes exhibited the smallest correlation with the phenotype after partialing out the root causal genes inferred by TWRCI. (D) More than 13% the causal variants exhibited horizontal pleiotropy. TWRCI annotated the remaining causal variants to eight gene expression levels. (E) TWRCI assigned approximately 78% of the causal variants to genes located on different chromosomes. Most causal variants annotated to a gene on the same chromosome fell within a one megabase distance from the TSS (blue, left). The average magnitude of the regression coefficients remained approximately constant with increasing distance from the TSS (red, right); the dotted line again corresponds to variants on different chromosomes. (F) The COPD-wide causal graph revealed multiple MHC class II genes as root causal. (G) UMAP dimensionality reduction revealed two clusters of COPD patients well-separated from the healthy controls. (H) The directed graphs highlighted different root causal genes within each of the two clusters.

We first compared the accuracy of the algorithms in variant annotation, graph reconstruction and CRCE estimation. We can compute the MACR metrics—representing two of the eleven evaluation criteria used in the previous section—with real data. We summarize the MACR for simultaneous variant annotation and graph reconstruction averaged over ten nested cross-validation folds in Fig 4A to assess algorithmic performance. TWRCI achieved the lowest MACR out of all combinations of algorithms within about 3 minutes (Fig C panels b and c in S1), indicating robust annotation and reconstruction. Performance differed primarily by the annotation method rather than the causal discovery algorithm. Conservative annotation algorithms, such as colocalization by SuSiE, again failed to achieve a low MACR because they frequently failed to annotate at least one variant to every gene expression level. MACR values for CRCE estimation followed a similar pattern (Fig 4B) because accurate annotation and reconstruction enabled accurate downstream CRCE estimation.

We next downloaded a set of silver standard genes enriched in genes that cause COPD [21,34]. The KEGG database does not contain a pathway for COPD, so we downloaded the gene set from the DisGeNet database instead (UMLS C0024117, curated) [35,36]. Many silver standard genes are causal but not *root* causal for COPD. If an algorithm truly identifies root causal genes, then partialing out the root causal genes from all of the downstream non-root causal genes and the phenotype should explain away the vast majority of the causal effect between the non-root causal genes and the phenotype according to the omnigenic root causal model. We therefore computed another MACR metric, the mean absolute correlation between the residuals of the silver standard genes and the residuals of the phenotype after partialing out the inferred root causal genes. TWRCI again obtained the lowest MACR value (Fig 4C). We conclude that TWRCI identified the root causal genes most accurately according to known causal genes in COPD.

#### Horizontal pleiotropy and trans-variants.

We studied the output of TWRCI in detail to gain insight into important issues in computational genomics. Previous studies have implicated the existence of widespread horizontal pleiotropy in many diseases [11]. TWRCI can annotate variants directly to the phenotype, so we can use TWRCI to assess the existence of widespread pleiotropy. The variable selection step of TWRCI identified fourteen gene expression levels surviving false discovery rate (FDR) correction at a liberal 10% threshold; eight of these levels ultimately caused the phenotype, including two psoriasis susceptibility genes, a complement protein and five MHC class II genes. Pairwise LD between the lead variants (those with the largest absolute debiased regression coefficients) for the five detected MHC genes was negligible (${\text{r}}^{2}<0.05$ for all pairs), and the maximum squared expression correlation was moderate (*r*^2^ = 0.44), confirming that these signals are not redundant due to genetic linkage or strong co-expression despite being located within the HLA region. TWRCI annotated 13.7% of the variants that cause COPD directly to the phenotype, despite competition for variants between the phenotype and the eight gene expression levels (Fig 4D). Many variants thus directly cause COPD by bypassing expression. We conclude that TWRCI successfully identified widespread horizontal pleiotropy in COPD. In contrast, cTWAS failed to identify any variants that bypass gene expression because all variants had very small effects on the phenotype, especially after accounting for gene expression; as a result, no variants ultimately had a posterior inclusion probability greater than 0.8 according to cTWAS.

TWRCI annotates both cis and trans-variants, so we examined the locations of the annotated variants relative to the TSS for each of the eight causal genes. Most of the variants lying on the same chromosome as the TSS fell within a one megabase distance from the TSS (Fig 4E blue). However, 78% of the variants were located on different chromosomes. We thus compared the variants annotated to causal genes by TWRCI against a previously published list of trans-eQTLs associated with any phenotype in a large-scale search [37] (Methods *Comparison to trans-eQTLs*). Variants annotated by TWRCI were located 1.94 times closer to trans-eQTLs than expected by chance (10,000 permutations, *p* < 0.001, 95% CI [1.93,1.95]). We next examined the effect sizes of the variants that cause the phenotype. We regressed the phenotype on variants inferred to directly or indirectly cause the phenotype using linear ridge regression. We then computed the moving average of the magnitudes of the regression coefficients over different distances from the TSS. The magnitudes remained approximately constant with increasing distance from the TSS (Fig 4E red). Moreover, the magnitudes for variants located on different chromosomes did not converge to zero (dotted line). We thus conclude that trans-variants play a significant role in modulating gene expression to cause COPD.

#### Root causal mechanism.

We next analyzed the output of TWRCI to elucidate the root causal mechanism of COPD. The pathogenesis of COPD starts with inhaled irritants that trigger an exaggerated and persistent activation of inflammatory cells such as macrophages, T cells and B cells [33]. These cells in turn regulate a variety of inflammatory mediators that promote alveolar wall destruction, abnormal tissue repair and mucous hypersecretion obstructing airflow. The root causal genes of COPD therefore likely involve genes mediating chronic and exaggerated inflammation in the lung.

Eight of the fourteen gene expression levels ultimately caused the COPD phenotype in the causal graph reconstructed by TWRCI (Fig 4F). The graph contained five MHC class II genes that present extracellular peptide antigens to CD4+ T cells in the adaptive immune response [38]. Subsequent activation of T cell receptors regulates a variety of inflammatory mediators and cytokines [39]. Moreover, the complement fragment C4a [40] as well as the psoriasis susceptibility genes PSORS1C1 and PSORS1C2 [41] help initiate and maintain the exaggerated inflammatory response seen in COPD. The recovered causal graph thus implicates chronic exaggerated inflammation as the root causal mechanism of COPD. TWRCI replicated these results by again discovering C4A and the MHC class II genes in an independent GWAS dataset composed of individuals of East Asian ancestry (Fig D panel a in S1).

We finally analyzed the personalized CRCE estimates in more detail. We can decompose the CRCE estimate of each gene into genetic and non-genetic components according to Eq (1). The genetic variants explained only 6.4% of the estimated variance of the CRCE for HLA-DRB5, 1.4% for C4A and <1% for the other six causal genes. We conclude that non-genetic factors account for nearly all of the explained variance in the CRCE estimates. We then performed UMAP dimensionality reduction [42] on the causal gene expression levels. Hierarchical clustering with Ward’s method [43] yielded three clear clusters of patients with COPD (Fig 4G) according to the elbow method on the sum of squares plot (Fig C panel a in S1). UMAP differentiated two of the COPD clusters from healthy controls, each with different mean CRCE estimates (Fig 4H directed graphs). For example, HLA-DRB5 had a large positive CRCE in cluster one but a large negative CRCE in cluster two. Note that the pink COPD cluster had many patients, but the blue and green clusters had a few patients, so we interpret their differences with caution. We conclude that the CRCE estimates differentiated patients into at least one subgroup consistent with the known pathobiology of COPD; we likewise obtained similar results in the second GWAS dataset (Fig D panels b and c in S1).

### Oxidative stress in ischemic heart disease

We also ran the algorithms on summary statistics of ischemic heart disease (IHD) consisting of 31,640 cases and 187,152 controls from Finland [44]. We used quality-controlled variant-expression-phenotype data of whole blood from GTEx [16] with 113 cases and 547 controls. We used whole blood because IHD arises from narrowing or obstruction of the coronary arteries most commonly secondary to atherosclerosis with transcription products released into the bloodstream [45]. We replicated the results using an independent set of GWAS summary statistics from 20,857 cases and 340,337 controls from the UK Biobank [46].

#### Accuracy.

**Fig 5:**
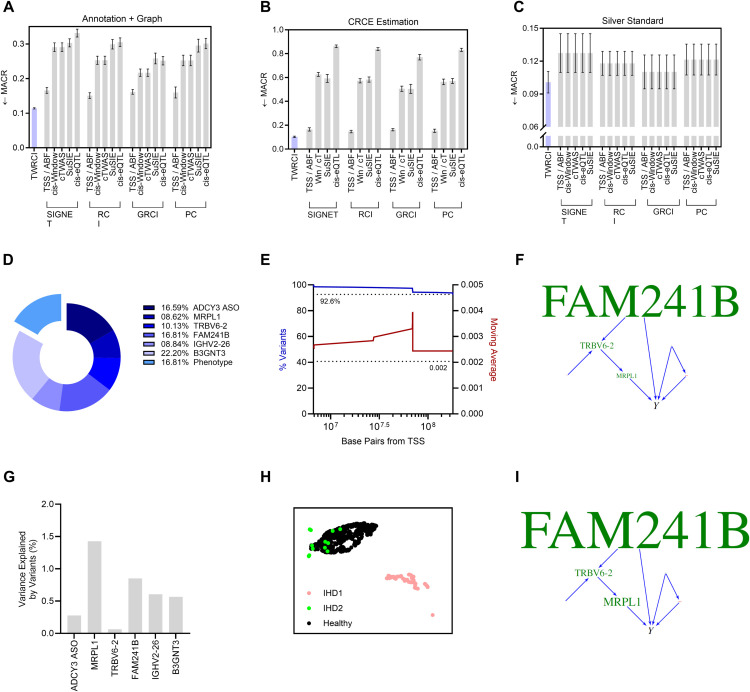
Results for IHD. (A) TWRCI again outperformed all other algorithms in combined annotation and graph reconstruction by achieving the lowest MACR. (B) TWRCI also estimated the CRCEs most accurately relative to all possible combinations of the other algorithms. (C) TWRCI outperformed all other algorithms with a silver standard set of genes causally involved in atherosclerosis. (D) TWRCI annotated variegated numbers of variants to six causal expression levels as well as the phenotype. (E) Nearly all of the annotated variants were located distal to the TSS (blue), and the magnitudes of their causal effects did not consistently increase or decrease on average with greater distance from the TSS (red). (F) TWRCI estimated the largest mean CRCEs for MRPL1, TRBV6-2 and FAM241B. (G) The annotated variants only explained a small proportion (<1.5%) of the variance for all CRCE estimates. (H) UMAP dimensionality reduction identified one cluster of patients clearly separated from healthy controls. (I) The mean CRCEs of MRPL1, TRBV6-2 and FAM241B remained the largest in this cluster.

We compared the algorithms in variant annotation, graph reconstruction and CRCE estimation accuracy. TWRCI achieved the lowest MACR in both cases (Fig 5A and B) within about one hour (Fig E panels b and c in S1). Cis-eQTLs and colocalization with SuSiE failed to annotate many variants because many trans-variants again predicted gene expression. We obtained similar results with a set of silver standard genes downloaded from the KEGG database (hsa05417) [47], where TWRCI outperformed all other algorithms (Fig 5C).

#### Horizontal pleiotropy and trans-variants.

The genetic variants predicted 27 gene expression levels at an FDR threshold of 10% with six genes inferred to cause the phenotype. We plot the six genes in the directed graph recovered by TWRCI in Fig 5F. TWRCI sorted approximately 8-23% of the causal variants to each of the six genes (Fig 5D). Moreover, TWRCI annotated approximately 17% of the causal variants directly to the phenotype supporting widespread horizontal pleiotropy in IHD. In contrast, cTWAS again did not detect any variants that directly cause the phenotype with a posterior inclusion probability greater than 0.8.

We analyzed the inferred causal effects of cis and trans-variants. Only 7.4% of the annotated variants were located on the same chromosome, and those on the same chromosome were often located over 10 megabases from the TSS (Fig 5E blue). Moreover, variants annotated by TWRCI were located 4.46 times closer to a published list of trans-eQTLs [37] than expected by chance (10,000 permutations, *p* = 0.0014, 95% CI [4.39,4.52]). The magnitudes of the regression coefficients remained approximately constant with increasing distance from the TSS and converged to 0.002—rather than to zero—on different chromosomes (Fig 5E red). We conclude that trans-variants also play a prominent role in IHD.

#### Root causal mechanism.

We next examined the root causal genes of IHD. IHD is usually caused by atherosclerosis, where sites of disturbed laminar flow and altered shear stress trap low-density lipoprotein (LDL) [48]. Reactive oxygen species then oxidize LDL and stimulate an inflammatory response. T cells in turn stimulate macrophages that ingest the oxidized LDL. The macrophages then develop into lipid-laden foam cells that form the initial fatty streak of an eventual atherosclerotic plaque. We therefore expect the root causal genes of IHD to involve oxidative stress and the inflammatory response.

TWRCI identified MRPL1, TRBV6-2 and FAM241B as the top three root causal genes (Fig 5F). MRPL1 encodes a mitochondrial ribosomal protein that helps synthesize complex proteins involved in the respiratory chain [49]. Deficiency of MRPL1 can lead to increased oxidative stress. TRBV6-2 encodes a T-cell receptor beta variable region involved in the inflammatory response and accumulation of T-cells in the atherosclerotic plaque [50]. Moreover, knocking out FAM241B induces the cytoplasmic buildup of large lysosome-derived vacuoles that generate foam cells [51]. We conclude that the root causal genes identified by TWRCI correspond to known genes involved in the pathogenesis of IHD. Finally, TWRCI rediscovered MRPL1 in a second independent GWAS dataset (Fig F panel a in S1).

We next dissected the CRCE estimates in detail. The annotated variants explained less than 1.5% of the CRCE variance for MRPL1, TRBV6-2 and FAM241B (Fig 5G). Non-genetic factors therefore account for the vast majority of the CRCE variance. UMAP dimensionality reduction and then hierarchical clustering on the causal genes discovered by TWRCI revealed two clusters of IHD patients (Fig E panel a in S1). The largest of the two clusters were distal to the cluster of healthy controls (Fig 5H). Furthermore, the FAM241B, TRBV6-2 and MRPL1 genes retained the largest mean CRCEs in this cluster (Fig 5I). TWRCI likewise replicated the large mean CRCE estimate for MRPL1 in the independent GWAS dataset (Fig F panels a and b in S1). We conclude that the CRCE estimates also identify genes that differentiate patient subgroups in IHD.

## Discussion

We introduced the CRCE of a gene, a measure of the causal effect of the genetic and non-genetic factors that directly cause a gene expression level on a phenotype. We then created the TWRCI algorithm that estimates the CRCE of each gene after simultaneously annotating variants and reconstructing the causal graph for improved statistical power. TWRCI annotates, reconstructs and estimates more accurately than alternative algorithms across multiple semi-synthetic and real datasets. Applications of TWRCI to COPD and IHD revealed succinct sets of root causal genes consistent with the known pathogenesis of each disease, which we verified by replication. Furthermore, clustering delineated patient subgroups whose pathogeneses were dictated by different root causal genes.

Our experimental results highlight the importance of incorporating trans-variants in statistical analysis. TWRCI annotated many variants distal to the TSS of each gene. These trans-variants improved the ability of the algorithm to learn models of gene regulation consistent with the correlations in the data according to the MACR criteria. Moreover, variants annotated by TWRCI were located closer to the positions of a previously published list of trans-eQTLs than expected by chance [37]. This enrichment is informative because it suggests that TWRCI is capturing the same underlying statistical signals identified by independent trans-eQTL studies. The excess proximity provides meaningful evidence that TWRCI detects real, biologically relevant trans-regulatory effects—especially since published trans-eQTLs are associated with diverse phenotypes and many disease-linked variants are known to act in trans, far from transcription start sites [52]. In contrast, nearest TSS, cis-windows, cTWAS, cis-eQTLs and the colocalization methods all rely on cis-variants that did not overlap with many GWAS hits both in the COPD and IHD datasets. Most GWAS hits likely lie distal to the TSSs in disease due to natural selection against cis-variants with large causal effects on gene expression [52]. As a result, algorithms that depend solely on cis-variants can fail to detect a large proportion of variants that cause disease in practice. Moreover, introducing a distance prior that favors cis-variants would further reduce sensitivity to functionally important trans-regulatory signals, degrading performance in disease-relevant applications.

TWRCI detected widespread horizontal pleiotropy accounting for 13-17% of the causal variants in both the COPD and IHD datasets. Previous studies have detected horizontal pleiotropy in around 20% of causal variants even after considering thousands of gene expression levels as well [11]. Moreover, many of the variants annotated to the phenotype by TWRCI correlated with gene expression (Figs C panel d and E panel d in S1). Accounting for widespread horizontal pleiotropy thus mitigates pervasive confounding between gene expression levels and the phenotype.

The cTWAS algorithm did not detect widespread pleiotropy in the real datasets. The algorithm also underperformed TWRCI in the semi-synthetic data, even when we restricted the analyses to variants that directly cause the phenotype. We obtained these results because cTWAS relies on the SuSiE algorithm to identify pleiotropic variants. However, pleiotropic variants usually exhibit weak causal relations to the phenotype, so most of these variants do not achieve a large posterior inclusion probability in practice. Algorithms that depend on *absolute* measures of certainty, such as posterior probabilities or p-values, miss many causal variants with weak causal effects in general. TWRCI therefore instead annotates variants by relying on *relative* certainty via a novel process called Competitive Regression, which we showed leads to more consistent causal models across multiple metrics.

We re-emphasize that TWRCI is the only algorithm that accurately recovers *root* causal genes *initiating* pathogenesis. Other methods such as colocalization and cTWAS identify causal genes *involved* in pathogenesis, regardless of whether the genes are root causal or not root causal. As a result, only TWRCI inferred a few genes with large CRCE magnitudes even in complex diseases. Moreover, genes with non-zero CRCE magnitudes explained away most of the causal effects of the non-root causal genes in the silver standards. Both of these results are consistent with the omnigenic root causal model, or the hypothesis that a small set of root causal genes drive the majority of pathogenesis in each patient even in complex disease by initiating widespread downstream gene expression changes [1].

Recall that the above root causal genes differ from driver genes and core genes. Root causal genes generalize driver genes by accounting for all of the factors that directly influence gene expression levels across all diseases, rather than just somatic mutations in cancer [3]. Accounting for both genetic and non-genetic factors is especially important when non-genetic factors explain the majority of the variance in the root causal effects, as we saw in COPD and IHD. Finally, root causal genes differ from core genes, or the gene expression levels that directly cause a phenotype, by focusing on the beginning rather than the end of pathogenesis [2]. Root causal genes may affect the expression levels of downstream genes so that many genes are differentially expressed between patients and healthy controls including many core genes. A few root causal genes can therefore increase the number of core genes.

TWRCI provably identifies root causal genes and attains high empirical accuracy, but the algorithm carries several limitations. Like most instrumental variable analysis algorithms such as the Transcriptome Wide Association Study [9] and Mendelian Randomization [10], TWRCI assumes that the phenotype is a variable with no downstream effects. We mitigate this by using tissue-specific gene expression measured in biologically relevant tissue for the disease, thereby increasing the plausibility that causal effects flow from expression to phenotype. While some authors have suggested that reverse causation is more likely to be detected using bidirectional approaches [53], it is important to recognize that reverse Transcriptome-Wide Mendelian Randomization (revTWMR) does not distinguish between direct and indirect genetic effects on the phenotype. By selecting variants that are marginally associated with the diagnosis as instruments, revTWMR may attribute apparent signals of reverse causality to variants whose effects are mediated upstream of the diagnosis, such as through gene expression or other intermediary pathways. This conflation of marginal association with direct causality is particularly problematic in the context of trans-eQTLs, which often act through complex, indirect, or pleiotropic mechanisms. This stands in contrast to the use of cis-eQTLs, which are more likely to exert direct regulatory effects on gene expression and, consequently, support more interpretable causal inference. As a result, even under ideal modeling conditions, revTWMR cannot reliably separate direct from indirect effects and therefore does not provide valid evidence for true reverse causation—particularly when the phenotype is a fixed diagnostic label, which likely serves as a terminal vertex in the underlying causal graph. Nevertheless, phenotypes can cause patients to change behaviors or take medications that in turn induce limited changes in gene expression, especially in blood [54].

The algorithm assumes an acyclic graph among gene expression levels and the phenotype, which may not reflect the reality of biological networks where feedback and dynamic cycles are common. While TWRCI can accommodate stationary cycles by transforming certain cyclic models into equivalent acyclic representations in the stationary regime [55], truly dynamic cycles in biology may not be fully captured by our current approach [56]. Additionally, TWRCI currently requires pre-filtering of variants, limiting scalability to genome-wide settings. Future work should focus on relaxing the single-DAG constraint by explicitly modeling bidirectional and feedback relations, potentially using single-cell or deconvoluted bulk RNA-seq data from both relevant and peripheral tissues. Further development is also needed to scale the method to millions of variants without pre-selection.

Despite these limitations, TWRCI addresses several challenges that confound other methods. First, the algorithm corrects for gene expression confounding through transcriptome-wide analysis and for genetic confounding arising from LD. This is a substantial advance over most existing methods, such as SuSiE, which only partially adjusts for confounders [24], and standard Mendelian Randomization approaches, which often overlooks LD structure and conditional associations [10,53]. TWRCI also sensitively detects horizontal pleiotropy, where many other approaches such as cTWAS lack practical power [21]. As a result, TWRCI demonstrates superior accuracy compared to prior methods, reflecting the value of its more flexible and comprehensive approach to confounding, pleiotropy, and stationary cycles. We therefore encourage the reader to view TWRCI as a practical generalization of standard causal inference frameworks for complex genomic settings.

In summary, we introduced an algorithm called TWRCI for accurate estimation and interpretation of the CRCE using personalized causal graphs. TWRCI empirically discovers only a few gene expression levels with large CRCE magnitudes even within different patient subgroups of complex disease in concordance with the omnigenic root causal model [57]. We conclude that TWRCI is a novel, accurate and disease agnostic procedure that couples variant annotation with graph reconstruction to identify root causal genes using observational data alone.

## Methods

We now provide detailed descriptions of the background, theory, algorithms, and experimental setups. TWRCI performs root causal inference, so we need to build the algorithm from exact, rigorous definitions to ensure that the method is not simply guided by heuristics but built on top of a robust causal discovery and inference framework.

### Background on causal discovery

Causal discovery refers to the process of discovering causal relations from data. We let italicized letters such as *Z*_*i*_ denote a singleton random variable and bold italicized letters such as Z denote sets of random variables. Calligraphic letters such as 𝒵 refer to sets of sets.

We consider a set of *p endogenous variables* Z. We represent a causal process over Z using a *structural equation model* (SEM) consisting of a series of deterministic functions:

$$Z_{i}=f_{i}(Pa(Z_{i}),E_{i})\;\forall Z_{i}\in Z,$$ (2)

where $Pa(Z_{i})\subseteq Z\;⧵\;Z_{i}$ denotes the *parents*, of direct causes, of *Z*_*i*_ and $E_{i}\in E$ an *exogenous variable*, also called an *error* or a *noise term*. We assume that the variables in E are mutually independent. The set $Ch(Z_{i})$ refers to the *children*, or direct effects, of *Z*_*i*_ where $Z_{j}\in \text{Ch}(Z_{i})$ if and only if $Z_{i}\in \text{Pa}(Z_{j})$.

We can associate an SEM with a *directed graph* 𝔾 by a drawing a directed edge from *Z*_*j*_ to *Z*_*i*_ when $Z_{j}\in \text{Pa}(Z_{i})$. We thus use the words *variable* and *vertex* interchangeably. A *root vertex* in 𝔾 refers to a vertex without any parents, whereas a *sink* or *terminal vertex* refers to a vertex without any children. A *path* between *Z*_0_ and *Z*_*n*_ corresponds to an ordered sequence of distinct vertices $\langle Z_{0},\ldots ,Z_{n}\rangle$ such that *Z*_*i*_ and *Z*_*i* + 1_ are adjacent for all 0≤i≤n−1. In contrast, a *directed path* from *Z*_0_ to *Z*_*n*_ corresponds to an ordered sequence of distinct vertices $\langle Z_{0},\ldots ,Z_{n}\rangle$ such that $Z_{i}\in \text{Pa}(Z_{i+1})$ for all 0≤i≤n−1. We say that *Z*_*j*_ is an *ancestor* of *Z*_*i*_, and likewise that *Z*_*i*_ is a *descendant* of *Z*_*j*_, if there exists a directed path from *Z*_*j*_ to *Z*_*i*_ (or $Z_{j}=Z_{i}$). We collect all ancestors of *Z*_*j*_ into the set $\text{Anc}(Z_{j})$, and all its non-descendants into the set $\text{Nd}(Z_{j})$. We write $Z_{i}\in \text{Anc}(A)$ when *Z*_*i*_ is an ancestor of any variable in A, and likewise Nd(A) for the non-descendants. The variable *Z*_*j*_ *causes Z*_*i*_ if *Z*_*j*_ is an ancestor of *Z*_*i*_ and $Z_{j}\text{⧸}=Z_{i}$. A *root cause* of *Z*_*i*_ corresponds to a root vertex that also causes *Z*_*i*_. This technical definition should not be confused with the colloquial use of “root cause,” which typically refers to a root vertex that causes *Z*_*i*_ *and* exerts a large detrimental causal effect on *Z*_*i*_; the term “detrimental” refers to promoting larger or smaller values of *Z*_*i*_, depending on whether larger or smaller values are interpreted as worse.

A *cycle* exists in 𝔾 when *Z*_*j*_ causes *Z*_*i*_ and vice versa. A *directed acyclic graph* (DAG) corresponds to a directed graph without cycles. A *collider* corresponds to *Z*_*j*_ in the triple $Z_{i}\to Z_{j}\leftarrow Z_{k}$. Two vertices *Z*_*i*_ and *Z*_*j*_ are d-connected given $W\subseteq Z\;⧵\;\{Z_{i},Z_{j}\}$ if there exists a path between *Z*_*i*_ and *Z*_*j*_ such that no non-collider is in W and all colliders are ancestors of W. We denote d-connection by $Z_{i}\text{⧸}⟂\;\;\;{⟂}_{d}Z_{j}|W$ for shorthand. The two vertices are *d-separated* given W, likewise denoted by $Z_{i}⟂\;\;\;{⟂}_{d}Z_{j}|W$, if they are not d-connected. The *Markov boundary* of *Z*_*i*_, denoted by $\text{Mb}(Z_{i})$, corresponds to the not necessarily unique but smallest set of variables in $Z\;⧵\;Z_{i}$ such that $Z_{i}⟂\;\;\;{⟂}_{d}(Z\;⧵\;\text{Mb}(Z_{i}))|\text{Mb}(Z_{i})$. A path is *blocked* by W if W contains at least one non-collider on the path or does not contain an ancestor of a collider (or both).

A probability density that obeys an SEM associated with the DAG 𝔾 also factorizes according to the graph:


$$p(Z)=\prod_{i=1}^{p}p(Z_{i}|\text{Pa}(Z_{i})).$$

Any density that factorizes as above obeys the *global Markov property*, where *Z*_*i*_ and *Z*_*j*_ are conditionally independent given W, or $Z_{i}⟂\;\;\;⟂Z_{j}|W$, if $Z_{i}⟂\;\;\;{⟂}_{d}Z_{j}|W$ [58]. A density obeys *d-separation faithfulness* when the converse holds: if $Z_{i}⟂\;\;\;⟂Z_{j}|W$, then $Z_{i}⟂\;\;\;{⟂}_{d}Z_{j}|W$. The Markov boundary of *Z*_*i*_ uniquely corresponds to the parents, children and parents of the children (or *spouses*) of *Z*_*i*_ under d-separation faithfulness.

### Causal modeling of variants, gene expression and the phenotype

**Fig 6:**
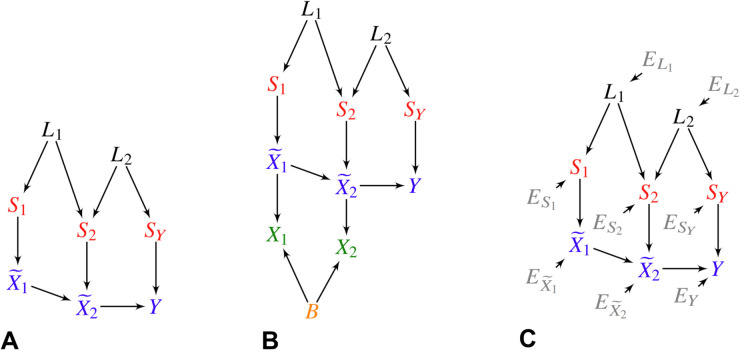
(A) An example of a DAG over Z. In (B), the additional vertices X denote counts corrupted by batch *B* effects and Poisson measurement error. (C) We can also augment the DAG in (A) with root vertex error terms E.

We divide the set of random variables Z into disjoint sets $Y\;\cup \;S\;\cup \;L\;\cup \;\tilde{X}$ corresponding to the phenotype *Y*, *q* genetic variants S, latent variables L modeling linkage disequilibrium (LD) and *m* gene expression levels $\tilde{X}$. We model the causal process over Z using the following SEM associated with a DAG 𝔾:

$$L_{i}=f_{i}(\text{Pa}(L_{i}),E_{i}),\;\forall L_{i}\in L\;S_{j}=f_{j}(\text{Pa}(S_{j}),E_{j}),\;\forall S_{j}\in S\;{\tilde{X}}_{k}=f_{k}(\text{Pa}({\tilde{X}}_{k}),E_{k}),\;\forall {\tilde{X}}_{k}\in \tilde{X},\;Y=f_{Y}(\text{Pa}(Y),E_{Y}),$$ (3)

where $\text{Pa}(L_{i})\subseteq L$, $\text{Pa}(S_{j})\subseteq L$, $\text{Pa}({\tilde{X}}_{k})\subseteq (\tilde{X}\;\cup \;S)$ and $\text{Pa}(Y)\subseteq (\tilde{X}\;\cup \;S)$ for any latent variable, any genetic variant, any gene expression level and the phenotype, respectively. In other words, linkage disequilibrium L generates variants S, and variants and gene expression generate other gene expression levels $\tilde{X}$ and the phenotype *Y* (example in Fig 6A). We assume that *Y* is a sink vertex, such that gene expression and variants cause *Y* but not vice versa.

Let $S_{i}$ denote the direct causes of ${\tilde{X}}_{i}$ in S. We require $S_{i}\text{⧸}=\emptyset$ for all ${\tilde{X}}_{i}\in \tilde{X}$ so that at least one variant directly causes each gene expression level. We also assume that any single variant can only *directly* cause one gene expression level or the phenotype (but not both). Investigators have reported only a few rare exceptions to this latter assumption in the literature, such as variants in super-enhancers or regulatory hubs [57,59,60]. A variant may however indirectly cause many gene expression levels.

We unfortunately cannot measure the exact values of gene expression using RNA sequencing (RNA-seq) technology. Numerous theoretical and experimental investigations have revealed that RNA-seq suffers from independent Poisson measurement error [61,62]:


$$X_{i}\sim \text{Pois}({\tilde{X}}_{i}\pi_{ij}),$$

where $\pi_{ij}$ denotes the *mapping efficiency* of ${\tilde{X}}_{i}$ in batch *j*. We thus sample Y∪S∪L∪X∪B from the DAG like the one shown in Fig 6B in practice, where *B* denotes the batch. With slight abuse of terminology, we will still call ${\tilde{X}}_{i}$ a *sink vertex* if it has only one child *X*_*i*_.

We can perform consistent regression under Poisson measurement error. Let $N=\sum_{i=1}^{m}X_{i}$ denote the library size and let ${\tilde{N}}_{j}=\sum_{i=1}^{m}{\tilde{X}}_{i}\pi_{ij}$ denote the true unobserved total gene expression level weighted by the mapping efficiencies in batch *j*. Also let $\tilde{U}\subseteq \tilde{X}$ and V⊆S refer to any subset of gene expression levels and variants, respectively. The following result holds:

**Lemma 1.** *Assume Lipschitz continuity of the conditional expectation for all* $N\geq n_{0}$:


$$𝔼\left|𝔼(Z_{i}|\tilde{U},V)-𝔼(Z_{i}|U,V,B)\right|\leq 𝔼C_{N}\left|\tilde{U}-\frac{U}{N}\frac{{\tilde{N}}_{B}}{\pi_{UB}}\right|,$$

*where $C_{N}\in O(1)$ is a positive constant, and we have taken an outer expectation on both sides. Then $𝔼(Z_{i}|\tilde{U},V)=\lim_{N\to \infty }𝔼(Z_{i}|U,V,B)$ almost surely.*

We delegate proofs to the Supplementary Materials S1. Intuitively, $\frac{U}{N}\frac{{\tilde{N}}_{B}}{\pi_{UB}}$ approaches $\tilde{U}$ as the library size increases, so the above lemma states that accurate estimation of $\tilde{U}$ implies accurate estimation of $𝔼(Z_{i}|\tilde{U},V)$. We can thus consistently estimate any conditional expectation $𝔼(Z_{i}|\tilde{U},V)$ using $𝔼(Z_{i}|U,V,B)$ when the library size approaches infinity. We only apply the asymptotic argument to bulk RNA-seq, where the library size is on the order of at least tens of millions. We henceforth implicitly assume additional conditioning on *B* whenever regressing to or on bulk RNA-seq data in order to simplify notation.

### Conditional root causal effects

We define the root causal effect of a gene expression level on the phenotype *Y*. We focus on Eq (3) with the endogenous variables Z and the exogenous variables E. If the error terms E are mutually independent, then we can *augment* the associated DAG 𝔾 with E by drawing a directed edge from each $E_{Z_{i}}$ to its direct effect *Z*_*i*_ (Fig 6C). We denote the resultant graph by ${𝔾}^{\prime }$, where we always have $E_{Z_{i}}\in {\text{Pa}}_{{𝔾}^{\prime }}(Z_{i})$ and the subscript emphasizes the augmented DAG; if we do not place a subscript, then we refer to the original DAG 𝔾. Only the error terms are root vertices in ${𝔾}^{\prime }$, so only exogenous variables that cause *Y* can be root causes of *Y*.

The *root causal effect* of *Z*_*i*_ on *Y* given the exogenous variables E is the causal effect of its direct causes in E on *Y*:

$$\mathbb{P}(Y|{\text{Pa}}_{{𝔾}^{\prime }}(Z_{i})\;\cap \;E)-\mathbb{P}(Y)=\mathbb{P}(Y|E_{Z_{i}})-\mathbb{P}(Y).$$ (4)

The variable *Z*_*i*_ is the first variable in Z affected by $E_{Z_{i}}$, and *Z*_*i*_ may in turn causally affect *Y*. The exogenous variable $E_{Z_{i}}$ models the effects of environmental, epigenetic and other *non-genetic* factors on *Z*_*i*_ because the set of endogenous variables $Z=Y\;\cup \;S\;\cup \;L\;\cup \;\tilde{X}$ includes the *genetic factors* S. The root causal effect is a special case of the *conditional root causal effect* (CRCE) given the exogenous variables E:


$$\mathbb{P}(Y|E_{Z_{i}},D)-\mathbb{P}(Y|D)$$

where (1) $D\subseteq {\text{Nd}}_{{𝔾}^{\prime }}(Z_{i})\;⧵\;(E_{Z_{i}}\;\cup \;Z_{i})$ and (2) $Y⟂\;\;\;{⟂}_{d}E_{Z_{i}}|Z_{i}\;\cup \;D$. The first condition ensures that D does not block any directed path from *Z*_*i*_ to *Y*. The second ensures that D eliminates any confounding between $E_{Z_{i}}$ and *Y*. The first condition actually implies the second in this case because E are root vertices. If we set D=∅, then we recover the unconditional root causal effect in Eq (4).

We are however interested in identifying the causal effects of both genetic *and* non-genetic factors on *Y* through gene expression $\tilde{X}$ with potential confounding between members of S due to LD. We therefore expand the set of exogenous variables to E∪S representing the non-genetic and genetic factors, respectively. We define the conditional root causal effect of ${\tilde{X}}_{i}\in \tilde{X}$ given the variables E∪S as:


$$\mathbb{P}(Y|{\text{Pa}}_{{𝔾}^{\prime }}({\tilde{X}}_{i})\;\cap \;(E\;\cup \;S),D)-\mathbb{P}(Y|D)\;=\;\mathbb{P}(Y|E_{i}\;\cup \;S_{i},D)-\mathbb{P}(Y|D),$$

where we write $E_{{\tilde{X}}_{i}}\;\cup \;S_{{\tilde{X}}_{i}}$ as $E_{i}\;\cup \;S_{i}$ to prevent cluttering of notation. The set $E_{i}\;\cup \;S_{i}$ thus refers to the direct causes of ${\tilde{X}}_{i}$ in E∪S. The above conditional root causal effect measures the causal effect of the root vertices E on *Y* as they pass through $E_{i}\;\cup \;S_{i}$ to ${\tilde{X}}_{i}$.

We can likewise choose any D such that $D\subseteq {\text{Nd}}_{{𝔾}^{\prime }}({\tilde{X}}_{i})\;⧵\;(E_{i}\;\cup \;S_{i}\;\cup \;{\tilde{X}}_{i})$ and $Y⟂\;\;\;{⟂}_{d}(E_{i}\;\cup \;S_{i})|{\tilde{X}}_{i}\;\cup \;D$. We choose D carefully to satisfy these two conditions as well as elicit favorable mathematical properties by setting $D={\tilde{V}}_{i}\;\cup \;(T\;⧵\;S_{i})$, where ${\tilde{V}}_{i}={\text{Pa}}_{{𝔾}^{\prime }}({\tilde{X}}_{i})\;\cap \;\tilde{X}$ and $T=\{S_{i}\in S:S_{i}\text{⧸}⟂\;\;\;{⟂}_{d}Y\}$. This particular choice of D allows us to write:


$$\Psi_{i}=\mathbb{P}(Y|E_{i}\;\cup \;S_{i},D)-\mathbb{P}(Y|D),\;=\mathbb{P}(Y|{\tilde{X}}_{i},D)-\mathbb{P}(Y|D),$$

so that we do not need to recover *E*_*i*_ as an intermediate step. We prove the second equality in Proposition 1 of the Supplementary Materials S1 under *exchangeability*, or no latent confounding by L between any two entries of $T\;\cup \;\{S_{i}\;⧵\;T:{\tilde{X}}_{i}\notin \text{Anc}(Y)\}$; this union corresponds to a set of sets including T and each entry of $\left\{S_{i}\;⧵\;T:{\tilde{X}}_{i}\notin \text{Anc}(Y)\right\}$ in the set. Exchangeability holds approximately in practice due to the weak causal relations emanating from variants to gene expression and the phenotype. Moreover, the assumption weakens with more variants in T. Now the first gene expression level in $\tilde{X}$ affected by $E_{i}\;\cup \;S_{i}$ is ${\tilde{X}}_{i}$. We thus call ${\tilde{X}}_{i}$ a *root causal gene* if ${\tilde{X}}_{i}$ also causes *Y* such that $\Psi_{i}\text{⧸}=0$.

We finally focus on the expected version of $\Psi_{i}$ to enhance computational speed, improve statistical efficiency and overcome Poisson measurement error according to Lemma 1:


$$\Gamma_{i}=𝔼(Y|{\tilde{X}}_{i},D)-𝔼(Y|D),$$

The *omnigenic root causal model* posits that |γ|≫0 for only a small subset of gene expression levels in each patient with Γ=γ. We thus seek to estimate the values *γ* for each patient. We use the acronym CRCEs to specifically refer to Γ from here on.

### Algorithm

#### Strategy overview.

We seek to accurately annotate, reconstruct and estimate the CRCEs using (1) summary statistics as well as (2) linked variant-expression-phenotype data. We summarize the proposed Transcriptome-Wide Root Causal Inference (TWRCI) algorithm in Algorithm 1. TWRCI first uses summary statistics to identify variants T associated with the phenotype at a liberal *α* threshold in Line 1. The algorithm also identifies gene expression levels R⊆X predictable by T in Line 1 from the variant-expression-phenotype data. TWRCI then annotates non-overlapping sets of variants to the phenotype in Line 2 and each gene expression level in Line 3 using a novel process called Competitive Regression; we prove that annotated variants include all of the direct causes in T. TWRCI arranges the gene expression levels in $\tilde{R}$ according to the causal order K during the annotation process. The algorithm finally recovers the directed graph uniquely given K in Line 4 and estimates the CRCE of each gene inferred to cause *Y* using the estimated graph $\hat{𝔾}$ and the annotations 𝒫 in Line 5. TWRCI can thus weigh and color-code each node in $\hat{𝔾}$ that causes *Y* by the CRCE estimates for each patient. We will formally prove that TWRCI is sound and complete at the end of this subsection.

**Algorithm 1 Transcriptome-Wide Root Causal Inference (TWRCI).**

**Input:** summary statistics, S ∪ X ∪ Y

**Output:** $𝒫,K,\hat{𝔾},\Gamma$

1: T,R,N← Variable selection with Algorithm 2

2: $P_{Y}\leftarrow$ Annotate some variants in T to *Y* using Algorithm 3

3: 𝒫,K← Annotate remaining variants in T to gene expression

levels and obtain the causal order using Algorithm 4

4: $\hat{𝔾}\leftarrow$ Recover DAG using Algorithms 5 and 6

5: Γ← Compute CRCE of each gene inferred to cause *Y* using $\hat{𝔾}$

and 𝒫

#### Variable selection.

We summarize the variable selection portion of TWRCI in Algorithm 2. TWRCI first reduces the number of variants using summary statistics by only keeping variants with a significant association to the phenotype at a very liberal *α* threshold (Line 1); we use 5e-5, or a three orders of magnitude increase from the usual threshold of 5e-8. We do not employ clumping or other pre-processing methods that may remove more variants from consideration because we are interested in resolving direct causal variants even within loci in high LD. Let T denote the variants that survive this screening step so that $T=\{S_{i}\in S:S_{i}\text{⧸}⟂\;\;\;{⟂}_{d}Y\}$.

The variable selection algorithm then identifies the gene expression levels predictable by T using the variant-expression-phenotype data in Line 2. We operationalize this step by linearly regressing X on T using half of the samples, and then testing whether the predicted level $\hat{X_{i}}$ and the true level *X*_*i*_ linearly correlate in the second half for each $X_{i}\in X$ [63]. This sample splitting procedure ensures proper control of the Type I error rate [64]. We keep gene expression levels R⊆X that achieve a q-value below a liberal FDR threshold of 10% [65]. We say that T is *relevant* if it contains at least one variant that directly causes each member of $\tilde{R}$. We finally repeat the above procedure after regressing out R from X⧵R and T in Line 3 in order to identify $\tilde{N}$, or all parents of $\tilde{R}$ in $\tilde{X}\;⧵\;\tilde{R}$. We call N the set of *nuisance variables*, since we will need to condition on them, but they do not contain the ancestors of *Y*. Algorithm 2 formally identifies the necessary ancestors needed for downstream inference:

**Lemma 2.** *Assume d-separation faithfulness and relevance. Then, (1) $T\;\cup \;\tilde{R}$ contains all of the ancestors of Y in $S\;\cup \;\tilde{X}$, and (2) $(\text{Mb}({\tilde{R}}_{i})\;\cap \;\tilde{X})\subseteq (\tilde{R}\;⧵\;{\tilde{R}}_{i})\;\cup \;\tilde{N}$ for any ${\tilde{R}}_{i}\in \tilde{R}$.*

**Algorithm 2 Variable selection.**

**Input:** summary statistics, S∪X∪Y

**Output:** T,R,N

1: $T\leftarrow S_{i}\in S$ such that $S_{i}\text{⧸}⟂\;\;\;⟂Y$ using summary statistics

2: $R\leftarrow X_{i}\in X$ such that $X_{i}\text{⧸}⟂\;\;\;⟂T$ using

variant-expression-phenotype data

3: $N\leftarrow X_{i}\in X\;⧵\;R$ such that $X_{i}\text{⧸}⟂\;\;\;⟂T|\tilde{R}$ using

variant-expression-phenotype data

#### Annotation for horizontal pleiotropy.

TWRCI next annotates the associated variants T to their direct effects in $\tilde{R}\;\cup \;Y$. The algorithm first annotates a sink vertex and then gradually works its way up the DAG until it annotates the final root vertex.

TWRCI assumes that *Y* is a sink vertex, so it first annotates to *Y*. A variant exhibits *horizontal pleiotropy* if it directly causes *Y*. We propose a novel Competitive Regression (CR) algorithm to annotate all members of $T\;\cap \;\text{Pa}(Y)=S_{Y}$ to *Y*.

We mildly assume equality in conditional expectation implies equality in conditional distribution and vice versa. Let $\tilde{Q}=\tilde{R}\;\cup \;Y$ and likewise Q=R∪Y. We also mildly assume that the following *contribution scores* exist and are finite: $\Delta_{ij}=𝔼|\partial 𝔼(Q_{i}|T)/\partial T_{j}|$ and $\gamma_{ij}=𝔼|\partial 𝔼(Q_{i}|\tilde{Q}\;⧵\;{\tilde{Q}}_{i},T)/\partial T_{j}|$. The scores correspond to the variable coefficients in linear regression.

We first provide the intuition behind the CR algorithm. If variant *T*_*j*_ *directly* causes *Y*, then it will predict *Y* given $T\;⧵\;T_{j}$ and *Y* given $T\;\cup \;\tilde{R}\;⧵\;T_{j}$ so that the respective regression coefficients satisfy $\Delta_{Yj}\text{⧸}=0$ and $\gamma_{Yj}\text{⧸}=0$. As a result, we have $|\Delta_{Yj}\gamma_{Yj}|>0$ in the ground truth. However, we need to set a threshold ϵ≥0 to determine if $|\Delta_{Yj}\gamma_{Yj}|>0$ when estimating $\left|\Delta_{Yj}\gamma_{Yj}\right|$ with finite samples because ${\hat{\Delta }}_{Yj}$ or ${\hat{\gamma }}_{Yj}$ (or both) are not exactly zero even when *T*_*j*_ does not directly cause *Y*. In other words, $|{\hat{\Delta }}_{Yj}{\hat{\gamma }}_{Yj}|\geq \epsilon$ but determining the best value of *ε* is non-trivial, especially in the non-parametric setting. CR avoids this issue by noting that, if *T*_*j*_ is a direct cause of *Y*, then *T*_*j*_ does not predict any gene expression level given $T\;⧵\;T_{j}$ so that $\max\Delta_{-Yj}^{2}=0$. CR therefore annotates *T*_*j*_ to *Y*, if $|{\hat{\Delta }}_{Yj}{\hat{\gamma }}_{Yj}|\geq \epsilon =\max{\hat{\Delta }}_{-Yj}^{2}$, i.e., ${\hat{\Delta }}_{Yj}{\hat{\gamma }}_{Yj}$ “beats” $\max{\hat{\Delta }}_{-Yj}^{2}$ in a competitive process, where $\max{\hat{\Delta }}_{-Yj}^{2}$ acts as an automatic threshold strictly greater than zero in the finite sample setting.

Formally, we use the contribution scores to annotate any $T_{j}\in T$ such that $|\Delta_{Yj}\gamma_{Yj}|\geq \max\Delta_{Rj}^{2}$ to *Y*, since this set of variants corresponds to a superset of $S_{Y}$ by the following result:

**Corollary 1.** *Under d-separation faithfulness, relevance and exchangeability, $|\Delta_{Yj}\gamma_{Yj}|\geq \max\Delta_{Rj}^{2}$ if and only if $T_{j}\notin Anc(R)$ or $T_{j}\in \text{Pa}(Y)$ (or both).*

The proof follows directly from Lemma 3 in the Supplementary Materials S1.

**Fig 7:**
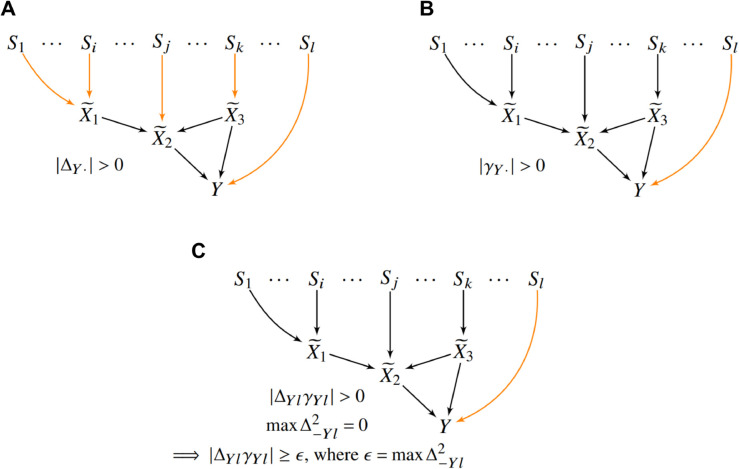
Detailed walkthrough of Competitive Regression using Fig 2C as an example. (A) Competitive Regression first regresses a terminal vertex *Y* on T and estimates the coefficients $\Delta_{Y\cdot }$ in Line 1. Any variant with a *ground truth* non-zero coefficient in $\Delta_{Y\cdot }$, such as $S_{1},S_{i},S_{j},S_{k}$ and *S*_*l*_ in the example, is a cause of *Y*. (B) The algorithm next regresses *Y* on T and the gene expression levels $\tilde{R}$ to estimate the coefficients $\gamma_{Y\cdot }$ in Line 2. Any variant with a ground truth non-zero coefficient in $\gamma_{Y\cdot }$ is a direct cause of *Y*, such as *S*_*l*_ in the example. As a result, we have $|\Delta_{Yl}\gamma_{Yl}|>0$ whenever *S*_*l*_ directly causes *Y*. (C) We unfortunately do not have access to the ground truth values but must estimate the coefficients $\Delta_{Y\cdot }$ and $\gamma_{Y\cdot }$ from data and set an appropriate threshold $|\Delta_{Yl}\gamma_{Yl}|\geq \epsilon$ to identify non-zero coefficients. Identifying an accurate threshold *ε* is difficult, so CR avoids this issue by setting $\epsilon =\max\Delta_{-Yl}^{2}$. In particular, if *S*_*l*_ is truly a direct cause of *Y*, then $\max\Delta_{-Yl}^{2}=0$ in the ground truth because *S*_*l*_ does not predict any member of $\tilde{R}$ conditional on $T\;⧵\;S_{l}$. As a result, we have the inequality $|\Delta_{Yl}\gamma_{Yl}|\geq \max\Delta_{-Yl}^{2}$, where $\epsilon =\max\Delta_{-Yl}^{2}$ acts as a data-driven threshold strictly greater than zero in the finite sample setting. TWRCI thus annotates *S*_*l*_ to *Y* in Line 3 when the inequality holds. Note that if both the left and right hand side of $|\Delta_{Yl}\gamma_{Yl}|\geq \max\Delta_{-Yl}^{2}$ are zero in the population setting, then *S*_*l*_ is not a direct cause of any member of $\tilde{R}\;\cup \;Y$. Assigning *S*_*l*_ to $P_{Y}$ this still yields a superset of the direct causes of *Y*. Finally, we can substitute *Y* in the above argument with any ${\tilde{X}}_{i}\in \tilde{R}$, so long as ${\tilde{X}}_{i}$ is a terminal vertex.

The CR algorithm summarized in Algorithm 3 computes the contribution scores in order to annotate variants to *Y*. Let $\Delta_{-i}$ denote the removal of the $i^{\text{th}}$ row from Δ corresponding to *Q*_*i*_ = *Y*. We use debiased linear ridge regression [66] to compute Δ in Line 1 and $\gamma_{i}$ in Line 2. Ridge regression is well-suited for high-LD settings as it enables unique and stable estimation of regression coefficients via a penalty term; however, this comes at the cost of shrinkage bias toward zero. The debiased ridge framework addresses this limitation by analytically removing the penalization-induced bias from the estimated coefficients. This ensures that the resulting estimates more accurately reflect true causal effects, rather than artifacts of regularization or collinearity. CR then compares the two quantities and outputs the set $P_{i}=\{T_{j}:|\Delta_{ij}\gamma_{ij}|\geq \max\Delta_{-ij}^{2}\}$, or a superset of $S_{i}\;\cap \;T$ not including any other variants with children in $\tilde{Q}\;⧵\;{\tilde{Q}}_{i}$ according to Corollary 1, in Line 3. We provide a step-by-step walkthrough of CR using an example in Fig 7.

**Algorithm 3 Competitive Regression (CR).**

**Input:** T, Q, N, ${\tilde{Q}}_{i}$

**Output:** $P_{i}$

1: Δ← Matrix of coefficients with rows obtained after

regressing *Q*_*j*_ on T for all $Q_{j}\in Q$

2: $\gamma_{i}\leftarrow$ Row vector of coefficients obtained after regressing *Q*_*i*_

on T and $(\tilde{Q}\;⧵\;{\tilde{Q}}_{i})\;\cup \;\tilde{N}$

3: $P_{i}\leftarrow \{T_{j}:|\Delta_{ij}\gamma_{ij}|\geq \max\Delta_{-ij}^{2}\}$

#### Annotation and causal order.

The CR algorithm requires the user to specify a known sink vertex. We drop this assumption by integrating CR into the Annotation and Causal Order (ACO) algorithm that automatically finds a sink vertex at each iteration.

ACO takes $R,N,Y,T,P_{Y}$ as input as summarized in Algorithm 4. The algorithm constructs a causal ordering over R∪Y in K by iteratively eliminating a sink vertex from R and appending it to the front of K. ACO also instantiates a list 𝒫 and assigns genetic variants $P_{i}=\{T_{j}:|\Delta_{ij}\gamma_{ij}|\geq \max\Delta_{-ij}^{2}\}\in 𝒫$ to each gene expression level $R_{i}\in R$ in Lines 8 and 18 using the following generalization of Corollary 1:

**Lemma 3.** *Assume d-separation faithfulness, relevance and exchangeability. Further assume that ${\tilde{Q}}_{i}$ is a sink vertex. Then, $|\Delta_{ij}\gamma_{ij}|\geq \max\Delta_{-ij}^{2}$ if and only if $T_{j}\notin Anc(Q\;⧵\;Q_{i})$ or $T_{j}\in Pa({\tilde{Q}}_{i})$ (or both).*

The set $P_{i}$ is thus again a superset of $S_{i}\;\cap \;T$, and any additional variants in $P_{i}$ do not directly cause another gene expression level or the phenotype.

ACO determines whether ${\tilde{R}}_{i}$ is indeed a sink vertex from data using the following result:

**Lemma 4.** ${\tilde{R}}_{i}$ *is a sink vertex if and only if $R_{i}⟂\;\;\;⟂(T\;⧵\;U_{i})|(\tilde{R}\;⧵\;{\tilde{R}}_{i})\;\cup \;\tilde{N}\;\cup \;U_{i}$ in Line 12 of ACO under d-separation faithfulness, relevance and exchangeability.*

ACO practically determines whether any ${\tilde{R}}_{i}$ is indeed a sink vertex post variable elimination by first computing the residuals *F*_*i*_ after regressing *R*_*i*_ on $\tilde{R}\;⧵\;{\tilde{R}}_{i}$, the nuisance variables $\tilde{N}$ and the identified variants $U_{i}$. A sink vertex ${\tilde{R}}_{i}$ has residuals *F*_*i*_ that are uncorrelated with the variants in $T\;⧵\;(O\;\cup \;U_{i})$ in Line 12 by Lemma 4, so ACO can identify the sink vertex ${\tilde{R}}_{i}$ in Line 15 as the variable with the smallest absolute linear correlation. The algorithm then appends *R*_*i*_ to the front of K and eliminates *R*_*i*_ from R in Lines 16 and 17, respectively. ACO finally adds $U_{i}$ to 𝒫 in Line 18, so $U_{i}$ can be removed from T of the next iteration through O. We formally prove the following result:

**Algorithm 4 Annotation and Causal Order (ACO).**

**Input:** R, N, Y, T, $P_{Y}$

**Output:** K,𝒫

1: 𝒫← Empty list

2: K←Y; $O\leftarrow P_{Y}$

3: **repeat**

4:   Δ← Contributions after regressing R on T⧵O

5:   C←∅

6:   **for all** $R_{i}\in R$ **do**

7:    $\gamma_{i}\leftarrow$ Contributions after regressing *R*_*i*_

on $\left(\tilde{R}\;⧵\;{\tilde{R}}_{i})\;\cup \;\tilde{N}\;\cup \;(T\;⧵\;O\right)$

8:    $U_{i}\leftarrow \{T_{j}:|\Delta_{ij}\gamma_{ij}|\geq \max\Delta_{-ij}^{2}\}$

9:    **if** $U_{i}=\emptyset$ **then**

10:     $C_{i}\leftarrow \infty$

11:    **else**

12:     $C_{i}\leftarrow$ Measure of dependence between *R*_*i*_ and $T\;⧵\;(O\;\cup \;U_{i})$

given $(\tilde{R}\;⧵\;{\tilde{R}}_{i})\;\cup \;\tilde{N}\;\cup \;U_{i}$

13:    **end if**

14:   **end for**

15:   $R_{i}\leftarrow$ Most independent variable in R according to C

16:   K← Append *R*_*i*_ to the front of K

17:   $R\leftarrow R\;⧵\;R_{i}$

18:   $P_{i}\leftarrow U_{i}$

19:   $O\leftarrow O\;\cup \;P_{i}$

20: **until** R=∅

**Algorithm 5 Graph discovery.**

**Input:** R, N, 𝒫, K, type I error rate *α*

**Output:** DAG $\hat{𝔾}$ over $\tilde{R}$

1: Form a fully connected undirected graph $\hat{𝔾}$ over $\tilde{R}$

2: l←−1

3: **repeat**

4:   Let *l* = *l* + 1

5:   **repeat**

6:    **for each** ${\tilde{R}}_{i}\in \tilde{R}$ **do**

7:     ${Adj}_{\hat{𝔾}}({\tilde{R}}_{i})\leftarrow$ Vertices adjacent to ${\tilde{R}}_{i}$ in $\hat{𝔾}$

8:    **end for**

9:    Select a new ordered pair of vertices $\left({\tilde{R}}_{i},{\tilde{R}}_{j}\right)$ that are

adjacent in $\hat{𝔾}$ and satisfy $|{Adj}_{\hat{𝔾}}({\tilde{R}}_{i})\;⧵\;{\tilde{R}}_{j}|\geq l$

10:    **repeat**

11:     Choose a new set $W\subseteq {Adj}_{\hat{𝔾}}({\tilde{R}}_{i})\;⧵\;{\tilde{R}}_{j}$ with |W|=l

12:     Test whether *R*_*i*_ and *R*_*j*_ are independent given

$\tilde{W}\;\cup \;\tilde{N}\;\cup \;P_{i}$ to obtain p-value *p*

13:     **if** p>α **then**

14:      Delete the edge ${\tilde{R}}_{i}-{\tilde{R}}_{j}$ from $\hat{𝔾}$

15:     **end if**

16:    **until** ${\tilde{R}}_{i}$ and ${\tilde{R}}_{j}$ are no longer adjacent in $\hat{𝔾}$ or all

such subsets with |W|=l have been considered

17:   **until** all ordered pairs of adjacent vertices $\left({\tilde{R}}_{i},{\tilde{R}}_{j}\right)$ in $\hat{𝔾}$

with $|{Adj}_{\hat{𝔾}}({\tilde{R}}_{i})\;⧵\;{\tilde{R}}_{j}|\geq l$ have been considered

18: **until** all pairs of adjacent vertices $\left({\tilde{R}}_{i},{\tilde{R}}_{j}\right)$ in $\hat{𝔾}$ satisfy

$|{Adj}_{\hat{𝔾}}({\tilde{R}}_{i})\;⧵\;{\tilde{R}}_{j}|\leq l$

19: Orient the edges of $\hat{𝔾}$ according to the causal order K

**Lemma 5.** *Under d-separation faithfulness, relevance and exchangeability, ACO recovers the correct causal order K over $\tilde{R}$ and $(S_{i}\;\cap \;T)\subseteq P_{i}$ for all $R_{i}\in \tilde{R}$.*

#### Causal graph discovery.

TWRCI uses the causal order K and the annotations 𝒫 to perform causal discovery. The algorithm runs the (stabilized) skeleton discovery procedure of the Peter-Clark (PC) algorithm to identify the presence or absence of edges between any two gene expression levels (Algorithm 5) [29,67]. We modify the PC algorithm so that it tests whether *R*_*i*_ and *R*_*j*_ are conditionally independent given $P_{i}\;\cup \;\tilde{N}$ and subsets of the neighbors of ${\tilde{R}}_{i}$ in $\tilde{R}\;⧵\;{\tilde{R}}_{i}$ in Line 12 to ensure that we condition on all parents of ${\tilde{R}}_{i}$. Finally, we orient the edges using the causal order K in Line 19 to uniquely recover the DAG over $\tilde{R}$:

**Lemma 6.** *Under d-separation faithfulness, relevance and exchangeability, the graph discovery algorithm outputs the true sub-DAG over $\tilde{R}$ given a conditional independence oracle, K and 𝒫.*

We next include the phenotype *Y* into the causal graph. We often only have a weak causal effect from gene expression and variants to the phenotype. We therefore choose to detect any causal relation to *Y* rather than just direct causal relations using Algorithm 6. Algorithm 6 only conditions on ${\tilde{V}}_{i}\;\cup \;P_{i}$ in Line 4 to discover both direct and indirect causation in concordance with the following result:

**Lemma 7.** *Under d-separation faithfulness, relevance and exchangeability, ${\tilde{R}}_{i}$ causes Y—and likewise the vertices $S_{i}\;\cup \;E_{i}$ cause Y—if and only if $Y\text{⧸}⟂\;\;\;⟂{\tilde{R}}_{i}|{\tilde{V}}_{i}\;\cup \;P_{i}.$*

**Algorithm 6 CRCE graph discovery.**

**Input:** R, N, Y, 𝒫, $\hat{𝔾}$ over $\tilde{R}$, type I error rate *α*

**Output:** DAG $\hat{𝔾}$ over $\tilde{R}\;\cup \;Y$

1: Add vertex *Y* in $\hat{𝔾}$

2: Draw a directed edge from each vertex in $\tilde{R}$ to Y in $\hat{𝔾}$

3: **for each** ${\tilde{R}}_{i}\in \tilde{R}$ **do**

4:   Test whether *R*_*i*_ and *Y* are independent given

${Pa}_{\hat{𝔾}}({\tilde{R}}_{i})\;\cup \;\tilde{N}\;\cup \;P_{i}$ to obtain p-value *p*

5:   **if** p>α **then**

6:    Delete the edge ${\tilde{R}}_{i}\to Y$ from $\hat{𝔾}$

7:   **end if**

8: **end for**

#### Conditional root causal effect estimation.

TWRCI finally estimates the CRCEs of the genes that cause *Y* given the recovered graph $\hat{𝔾}$ and the annotations 𝒫. We estimate the two conditional expectations in Eq (5) using kernel ridge regression [68]. We embed *X*_*i*_ and ${Pa}_{\hat{𝔾}}({\tilde{R}}_{i})={\tilde{V}}_{i}$ using a radial basis function kernel but embed $T\;⧵\;P_{i}$ using a normalized linear kernel. We normalize the latter to prevent the linear kernel from dominating the radial basis function kernel, since the variables in $T\;⧵\;P_{i}$ typically far outnumber those in ${\tilde{V}}_{i}$.

We now integrate all steps of TWRCI by formally proving that TWRCI is sound and complete:

**Theorem 1.** *(Fisher consistency) Under d-separation faithfulness, relevance and exchangeability, TWRCI identifies all of the direct causal variants of $Y\;\cup \;(Anc(Y)\;\cap \;\tilde{X})$, the unique causal graph over $Y\;\cup \;(Anc(Y)\;\cap \;\tilde{X})$ and the CRCEs of $Anc(Y)\;\cap \;\tilde{X}$ almost surely as N→∞ with Lipschitz continuous conditional expectations and a conditional independence oracle.*

We perform conditional independence testing by correlating the regression residuals of smooth non-linear transformations of the gene expression levels and phenotype [69]. As a result, Lemma 1 also enables accurate conditional independence testing over subsets of $T\;\cup \;\tilde{R}\;\cup \;\tilde{N}$, even though we only have access to T∪R∪N.

#### Time complexity.

We analyze the time complexity of TWRCI in detail. TWRCI can admit different regression procedures, so we will assume that each regression takes $\text{O}({\text{c}}^{3})$ time, where *c* denotes the dimensionality of the conditioning set typically much larger than the sample size *n*. Most regression procedures satisfy the requirement.

TWRCI first runs Algorithm 2 which requires *O*(*q*) time in Line 1 with summary statistics, $\text{O}({\text{q}}^{3}\text{m})$ time in Line 2 with at most *m* regressions on T, and $O(m^{3}(m\;+\;q))$ time for at most *m* + *q* regressions on $\tilde{R}$ in Line 3. Algorithm 2 thus takes $O(m^{4}\;+\;m^{3}q)\;+\;O(q^{3}m)$ time in total.

TWRCI next annotates to *Y* using Algorithm 3 which takes $O(q^{3}m)\;+\;O((m+q{)}^{3})$ time for Lines 1 and 2, respectively. Annotation to *Y* therefore carries a total time complexity of $\text{O}({\text{m}}^{3}{\text{q}}^{3})$. TWRCI then runs Algorithm 4. Each iteration of the repeat loop in Line 3 of Algorithm 4 takes $\text{O}({\text{q}}^{3})$ time for the regression in Line 4 and $O(m(m\;+\;q{)}^{3})$ time for the at most *m* regressions in Line 7. The repeat loop iterates at most *m* times, so Algorithm 4 has a total time complexity of $O(m(q^{3}\;+\;m(m\;+\;q{)}^{3}))=O(m^{5}q^{3})$.

Algorithm 5 dominates Algorithm 6 in time during the causal graph discovery portion of TWRCI. Algorithm 5 runs in $O(m^{e}(m\;+\;q{)}^{3})=O(m^{e+3}q^{3})$ time, where *e* denotes the maximum neighborhood size [29]. Finally, CRCE estimation in Line 5 requires $O(2m(m\;+\;q{)}^{3})=O(m^{4}q^{3})$ time for at most 2*m* regressions on expression levels and variants. Thus TWRCI in total requires $O(m^{4}\;+\;m^{3}q)\;+\;O(q^{3}m)\;+\;O(m^{3}q^{3})\;+\;O(m^{5}q^{3})\;+\;O(m^{e+3}q^{3})\;+\;O(m^{4}q^{3})=O(m^{5}q^{3})\;+\;O(m^{e+3}q^{3})$ time. We conclude that the ACO and Graph Discovery sub-algorithms dominate the time complexity of TWRCI. We list empirical runtime results in Supplementary Materials S1.

### Comparators

We compared TWRCI against state of the art algorithms enumerated below.

Annotation:

1. Nearest TSS: annotates each variant to its closest gene according to the TSS.
2. Cis-window: annotates a variant to a gene if the variant lies within a one megabase window of the TSS. If a variant lies in multiple windows, then we assign the variant to the closest TSS.
3. Causal transcriptome-wide association study (cTWAS) [21]: annotates variants to genes using cis-windows and then accounts for horizontal pleiotropy using the Sum of Single Effects (SuSiE) algorithm.
4. Cis-eQTLs [22]: annotates a variant to a gene if (1) the variant lies in the cis-window of the gene per above, and (2) the variant correlates most strongly with that gene expression level relative to the other levels.
5. Colocalization with approximate Bayes factors [23]: annotates each variant to the gene expression level with the highest colocalization probability according to approximate Bayes factors. We *could not* differentiate this method from cis-windows using the MACR criteria for the real data (Methods *Metrics*), since the algorithm always assigns higher approximate Bayes factors to cis-variants.
6. Colocalization with SuSiE [23,24]: same as above but with probabilities determined according to SuSiE. We *could* differentiate this method from cis-windows using the MACR criteria for the real data.

Causal Graph Reconstruction:

1. SIGNET [25,26]: predicts gene expression levels from variants using ridge regression and then recovers the genetic ancestors of each expression level by running the adaptive LASSO on the predicted expression levels. The method thus assumes linearity.
2. RCI [27]: assumes a linear non-Gaussian acyclic model [70], and recovers the causal order by maximizing independence between gene expression level residuals obtained from linear regression.
3. GRCI [28]: same as above but assumes an additive noise model [71] and uses non-linear regression.
4. PC/CausalCell [30]: runs the stabilized PC algorithm [29,67] on the gene expression levels using a non-parametric conditional independence test [69].

### Semi-synthetic data

The causal graph reconstruction algorithms all require a variable selection step with gene expression data, since they cannot scale to the tens of thousands of genes with the neighborhood sizes seen in practice [1,30]. We therefore assessed the performance of the algorithms independent of variable selection by first instantiating a DAG directly over $\tilde{Q}$ with *p* = 30 variables including 29 gene expression levels and a single phenotype. We generated a linear SEM obeying Eq (3) such that ${\tilde{Q}}_{i}=\tilde{Q}\beta_{i}\;+\;S_{i}\theta_{i}\;+\;E_{i}$ for every ${\tilde{Q}}_{i}\in \tilde{Q}$ with $E_{i}\sim 𝒩(0,1/25)$ to enable detection of weak causal effects from variants. We drew the coefficient matrix *β* from a Bernoulli(2/(p−1)) in the upper triangular portion of the matrix and then randomly permuted the ordering of the variables. The resultant DAG has an expected neighborhood size of 2. We then weighted the coefficient matrix between the gene expression levels and phenotype by sampling uniformly from [−1,−0.25]∪[0.25,1]. This process ensures gene expression levels are interdependent, mimicking biological regulatory relationships.

We instantiated the variants T and *θ* as follows. We downloaded summary statistics from a wide variety of IEU datasets listed in Table 1 and filtered variants at a liberal *α* threshold of 5e-5. We selected a variant to be closest to the TSS of each gene uniformly at random and assigned direct causal variants to the 29 gene expression levels with probability proportional to the inverse of the absolute distance from the closest variant plus one. As a result, variants closer to the TSS are more likely to have a direct causal effect on the gene expression level. We assigned the remaining variants to the phenotype. We sampled T by bootstrap from the GTEx version 8 [16] individual-level genotype data to preserve realistic LD patterns. We sampled the weights *θ* uniformly from [−0.15,−0.05]∪[0.05,0.15] because variants usually have weak causal effects.

**Table 1:** Variant data used during semi-synthetic data generation.

Dataset	Trait	# Variants	# Cases	# Controls
ieu-b-5067	Alzheimer’s	669	954	487331
ieu-b-4967	Appendicitis	736	4604	481880
ieu-b-4972	Endocarditis	300	1080	485404
ieu-b-4971	Cholecystitis	691	4052	482432
ieu-b-4973	Lower respiratory tract infection	1116	14135	472349
ieu-a-1187	Major depression	566	135458	344901
ieu-b-4965	Colorectal cancer	1791	5657	372016
ieu-b-5063	Upper respiratory tract infection	451	2795	483689
ieu-b-4956	Lymphoid leukemia	988	760	372016
ieu-b-4953	Liver cell carcinoma	517	168	372016

We converted the above linear SEM to a non-linear one by setting ${\tilde{Q}}_{i}\leftarrow softplus({\tilde{Q}}_{i})=ln(1\;+\;exp({\tilde{Q}}_{i}))$ for each ${\tilde{Q}}_{i}\in \tilde{Q}$. We obtained each measurement error corrupted surrogate *R*_*i*_ by sampling from $Pois({\tilde{R}}_{i}\pi_{i1})$ for each ${\tilde{R}}_{i}\in \tilde{R}$. We drew the mapping efficiencies $\pi_{\cdot 1}$ for a single batch from the uniform distribution between 100 and 10000 for the bulk RNA sequencing data. We repeated the entirety of the above procedure 100 times to generate 100 independent variant-expression-phenotype datasets. We ran TWRCI and all combinations of the comparator algorithms on each dataset.

### Real data

#### Quality control.

We selected variants T at an *α* threshold of 5e-5 for both the COPD and IHD summary statistics. We harmonized the variant data of the IEU and GTEx datasets by lifting the GTEx variant data from the hg38 to hg19 build using the liftover command in BCFtools version 1.18 [72]. We ensured that the reference and alternative alleles matched in both datasets after lifting for every variant. We removed gene expression levels with a mean count of less than five. We subjected the gene expression data to an inverse hyperbolic sine transformation to mitigate the effects of outliers. We regressed out the first 5 principal components, sequencing platform (Illumina HiSeq 2000 or HiSeq X), sequencing protocol (PCR-based or PCR-free) and sex from all variables in the linked GTEx variant-expression-phenotype data. Then, we either included age as a covariate for algorithms that accept a nuisance covariate, or regressed out age from the expression and phenotype data for algorithms that do not accept a nuisance covariate.

#### Comparison to trans-eQTLs.

TWRCI annotated many trans-variants in both of the real datasets. Other authors have proposed *trans-eQTLs* as variants that lie distal to the TSS and correlate with at least one reported phenotype in the Catalog of Published GWAS [73]. TWRCI annotates variants based on direct causality rather than correlation and an overlap with another phenotype. However, we hypothesized that the variants discovered by TWRCI should still lie close to at least a subset of the trans-eQTLs. To test this hypothesis, we downloaded trans-eQTL results from the eQTLGen database [37]. We then standardized the positions of the variants within each chromosome by their standard deviation to account for variable chromosome length and polymorphism density. Next, we computed the nearest neighbor distances between the variants annotated to causal genes by TWRCI and the trans-eQTLs. We used the median of these normalized distances *M* as a robust statistic of central tendency.

We used a permutation test to test the null hypothesis that the variants annotated to causal genes by TWRCI are distributed arbitrarily far from the trans-eQTLs. We recomputed the median statistic 10,000 times after permuting the positions of the trans-eQTL variants. The p-value corresponds to the proportion of permuted statistics smaller than *M*. We reject the null hypothesis—and thus conclude that the variants annotated to causal genes by TWRCI lie close to trans-eQTLs—when the p-value falls below 0.05.

### Metrics

We evaluated the accuracy of the algorithms using the eleven metrics listed below for the synthetic data. We evaluated annotation quality using the following four metrics:

1. Matthew’s Correlation Coefficient (MCC) [74] between the estimated annotations and the ground truth direct causal variants. Larger is better.
2, 3. Precision and recall. Larger is better.
4. Rank of the estimated coefficients $\hat{\theta }$ normalized by the rank of the ground truth coefficients *θ*. Larger is better.

We also computed the above four quantities only using the variants that directly cause the phenotype in order to evaluate the ability of the algorithms to account for horizontal pleiotropy. We evaluated the causal graph reconstruction quality using the following four metrics:

5. Structural Hamming Distance (SHD) [75] between the estimated and the ground truth causal graph. Smaller is better.
6. MCC between the estimated and the ground truth causal graph. Larger is better.
7, 8. Precision and recall. Larger is better.

We evaluated combined annotation and graph reconstruction quality using Lemma 4:

9. Mean absolute correlation of the residuals (MACR) defined as the mean absolute correlation between (a) the variants T and ancestral gene expression levels, and (b) the gene expression residuals after partialing out the inferred parents. Smaller is better under the global Markov property and exchangeability. If the algorithm infers no direct causal variants in T and no parents in $\hat{𝔾}$ for some ${\tilde{R}}_{i}$, then this situation violates the relevance assumption, where at least one variant in T directly causes ${\tilde{R}}_{i}$. We thus set the absolute correlation of ${\tilde{R}}_{i}$ to one in this case.

We assessed the accuracy in CRCE estimation using the following metrics:

10. Root mean squared error between the estimated CRCE and the ground truth CRCE averaged over all gene expression levels. We do not have access to the ground truth CRCE, so we estimate it to negligible error with kernel ridge regression using the ground truth parents. Smaller is better.
11. MACR between (a) the residuals $Y-\hat{𝔼}(Y|{\tilde{R}}_{i},D)$ and (b) the inferred set $P_{i}$, which should be zero under the global Markov property and exchangeability. Smaller is better. We again set the absolute correlation to one for ${\tilde{R}}_{i}$ if the algorithm infers no direct causal variants and no parents in $\hat{𝔾}$ under relevance.

We can compute the MACR metrics 9. and 11. on real data, so we evaluate the algorithms using these two metrics in the IHD and COPD datasets. We also have access to silver standard sets of genes known to be causally involved in disease from either the DisGeNet [36] or KEGG database [47]. We therefore compute a third MACR metric with the real data:

12. A causal gene should at least correlate with the phenotype, so we first correlate the silver standard genes with the phenotype and only keep silver standard genes with a significant correlation (*p* < 0.05 uncorrected). We then compute a MACR metric between (a) the kept silver standard genes after partialing out genes with non-zero CRCEs and (b) the phenotype after partialing out genes with non-zero CRCEs.

## Supporting information

S1 Supplementary Materials Extended results, replications, and proofs.
(PDF)
