# Correction: Transcriptome-wide root causal inference

**DOI:** 10.1371/journal.pcbi.1013979

**Published:** 2026-02-18

**Authors:** The PLOS Computational Biology Staff

## Notice of Republication

This article was republished on January 21, 2026, to correct errors that were introduced during the typesetting process. There was an error within the introduction where reference 20 was incorrectly formatted within the text. There was an error within the XML of the paper where a proof comment was erroneously kept. S1 Supplementary Materials is omitted from the list of Supporting Information. It can be viewed below.

The publisher apologizes for the errors. Please download this article again to view the correct version. The originally published, uncorrected article and the republished, corrected articles are provided here for reference.

## Supporting information

S1 Supplementary Materials. Extended results, replications, and proofs.
(PDF)

S1 File Originally published, uncorrected article. (PDF)

S2 File Republished, corrected article. (PDF)
