# Supplementary material for: Transcriptome-wide root causal inference
Source: PLoS Comput Biol. 2025 Sep 2;21(9):e1013461. doi: 10.1371/journal.pcbi.1013461 (PMC12413095; doi:10.1371/journal.pcbi.1013461)
Supplement: S1 Supplementary Materials — Extended results, replications, and proofs. (PDF) [file pcbi.1013461.s001.pdf]

A Supplementary Materials

A.1 Additional Semi-Synthetic Data Results

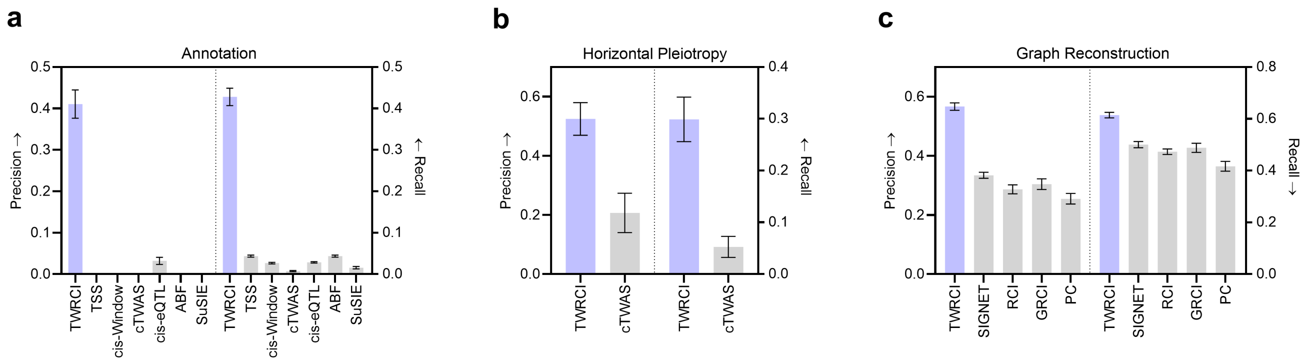

Fig A. Precision and recall results for the semi-synthetic datasets. TWRCI achieved the highest precision and recall for (a) direct causal annotation, (b) annotation focused on horizontal pleiotropy only, and (c) graph reconstruction.

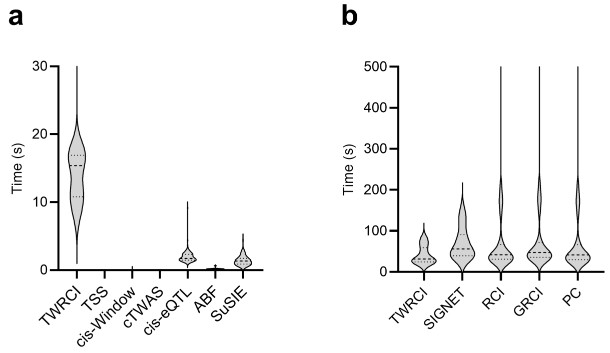

Fig B. Timing results for the semi-synthetic datasets split into the variant annotation and graph reconstruction portions because they took the longest by far. (a) TWRCI took the longest time during annotation, but (b) all algorithms spent the majority of the time in causal graph reconstruction over $\widetilde{\boldsymbol{R}}$ in congruence with the time complexity results of Methods *Time Complexity*. TWRCI completed within about 3 minutes overall.

A.2 Additional COPD Data Results

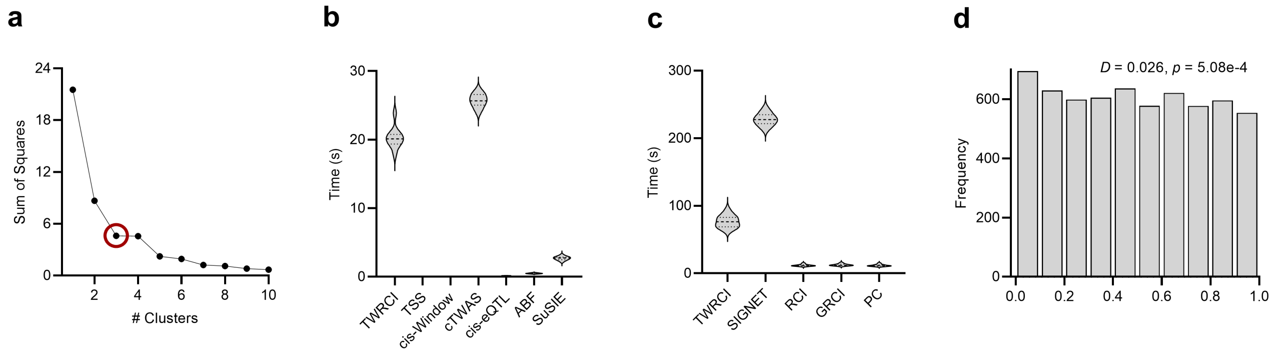

Fig C. Additional results for COPD. (a) Sum of squares plot for hierarchical clustering using Ward's method revealed three clusters according to the elbow method, or the cluster size with the maximum distance from the imaginary line drawn between the first and last cluster sizes. TWRCI took the second longest time to complete in annotation (b) and the second longest time to complete in graph reconstruction (c). RCI, GRCI and PC all took a much smaller amount of time to reconstruct the causal graph because they ignore the genetic variants. (d) Histogram of Pearson correlation test p-values computed between variants annotated to the phenotype and gene expression levels. The p-values did not follow a uniform distribution according to the Kolomogorov-Smirnov test with statistic D indicating the presence of confounding between the variants annotated to the phenotype and gene expression.

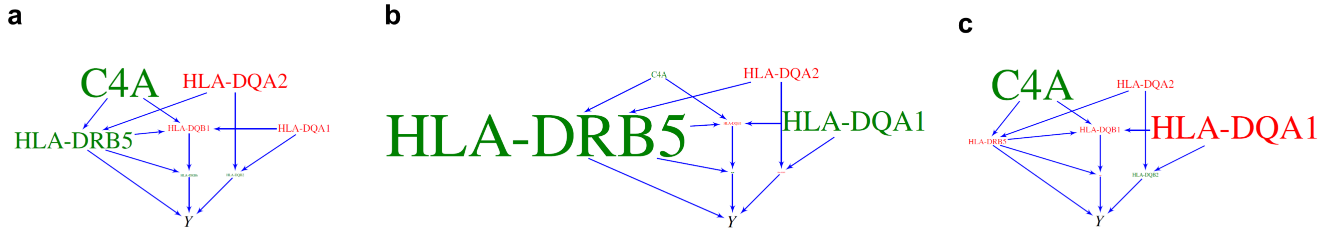

Fig D. Replication results in an independent set of individuals of East Asian ancestry (dataset ebi-a-GCST90018587). We summarize results for (a) all patients, (b) cluster one in Fig 4 (g) and (c) cluster two. TWRCI again identified C4A and multiple MHC class II genes involving the adaptive immune system.

A.3 Additional IHD Data Results

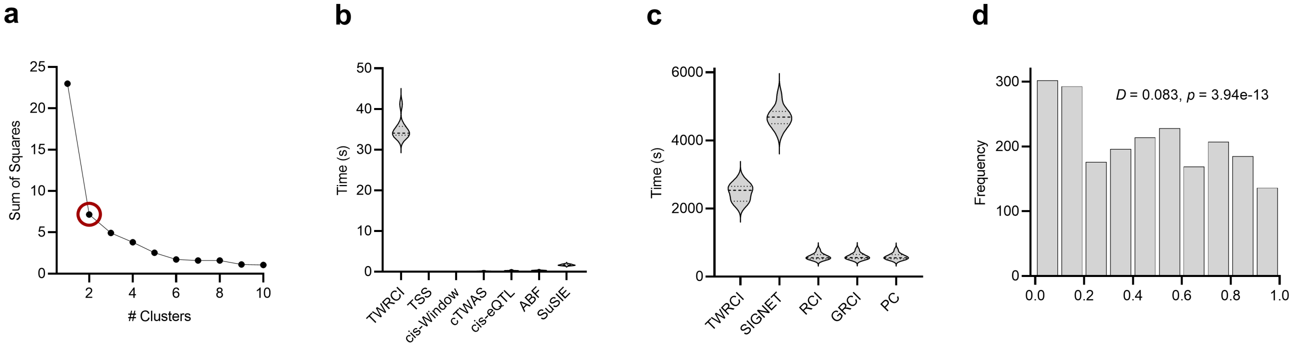

Fig E. Additional results for IHD. (a) Sum of squares plot revealed two clusters according to the elbow method. (b) TWRCI took the longest to annotate, but (c) the timing results for graph reconstruction dominated in this case. Methods using SIGNET thus took the longest overall in this dataset. (d) Histogram of Pearson correlation test p-values were again non-uniform, indicating confounding between the variants annotated to the phenotype and gene expression.

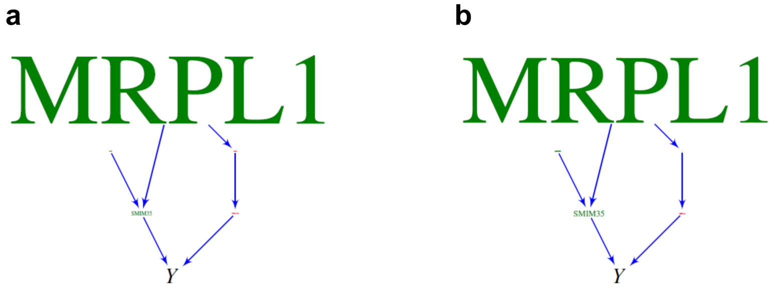

Fig F. Replication results in an independent set of patients from the UK Biobank (dataset ukb-d-I9_IHD). We summarize results for (a) all patients, and (b) cluster one. TWRCI again identified MRPL1 as a root causal gene with a large positive CRCE.

A.4 Proofs

Lemma 1. *Assume Lipschitz continuity of the conditional expectation for all $N \geq n_0$:*

$$\mathbb{E}\Big|\mathbb{E}(Z_i|\widetilde{\boldsymbol{U}},\boldsymbol{V}) - \mathbb{E}(Z_i|\boldsymbol{U},\boldsymbol{V},B)\Big| \leq \mathbb{E}C_N\left|\widetilde{\boldsymbol{U}} - \frac{\boldsymbol{U}}{N}\frac{\widetilde{N}_B}{\pi_{\boldsymbol{U}B}}\right|,$$

where $C_N \in O(1)$ is a positive constant, and we have taken an outer expectation on both sides. Then $\mathbb{E}(Z_i|\widetilde{\boldsymbol{U}},\boldsymbol{V}) = \lim_{N\to\infty}\mathbb{E}(Z_i|\boldsymbol{U},\boldsymbol{V},B)$ almost surely.

Proof. We can write the following sequence:

$$\begin{aligned}
&\mathbb{E}\Big|\mathbb{E}(Z_i|\widetilde{\boldsymbol{U}},\boldsymbol{V}) - \lim_{N\to\infty}\mathbb{E}(Z_i|\boldsymbol{U},\boldsymbol{V},B)\Big| = \mathbb{E}\lim_{N\to\infty}\Big|\mathbb{E}(Z_i|\widetilde{\boldsymbol{U}},\boldsymbol{V}) - \mathbb{E}(Z_i|\boldsymbol{U},\boldsymbol{V},B)\Big| \\
\leq\; &\mathbb{E}\lim_{N\to\infty}C_N\left|\widetilde{\boldsymbol{U}} - \frac{\boldsymbol{U}}{N}\frac{\widetilde{N}_B}{\pi_{\boldsymbol{U}B}}\right| \leq \mathbb{E}C\left|\widetilde{\boldsymbol{U}} - \lim_{N\to\infty}\frac{\boldsymbol{U}}{N}\frac{\widetilde{N}_B}{\pi_{\boldsymbol{U}B}}\right| = \mathbb{E}C\left|\widetilde{\boldsymbol{U}} - \frac{\widetilde{\boldsymbol{U}}\pi_{\boldsymbol{U}B}}{\widetilde{N}_B}\frac{\widetilde{N}_B}{\pi_{\boldsymbol{U}B}}\right| = C\mathbb{E}\Big|\widetilde{\boldsymbol{U}} - \widetilde{\boldsymbol{U}}\Big| = 0,
\end{aligned}$$

where we have applied the Lipschitz continuity assumption at the first inequality. We have $C_N \leq C$ for all $N \geq n_0$ in the second inequality because $C_N \in O(1)$. With the above bound, choose $a > 0$ and invoke the Markov inequality:

$$\mathbb{P}\left(\Big|\mathbb{E}(Z_i|\widetilde{\boldsymbol{U}},\boldsymbol{V}) - \lim_{N\to\infty}\mathbb{E}(Z_i|\boldsymbol{U},\boldsymbol{V},B)\Big| \geq a\right) \leq \frac{1}{a}\mathbb{E}\Big|\mathbb{E}(Z_i|\widetilde{\boldsymbol{U}},\boldsymbol{V}) - \lim_{N\to\infty}\mathbb{E}(Z_i|\boldsymbol{U},\boldsymbol{V},B)\Big| = 0.$$

The conclusion follows because we chose a arbitrarily. ☐

Proposition 1. *We have $\mathbb{P}(Y|E_i \cup \boldsymbol{S}_i, \boldsymbol{D}) - \mathbb{P}(Y|\boldsymbol{D}) = \mathbb{P}(Y|\widetilde{X}_i, \boldsymbol{D}) - \mathbb{P}(Y|\boldsymbol{D})$ under exchangeability.*

Proof. We can write:

$$\begin{aligned}
&\mathbb{P}(Y|E_i \cup \boldsymbol{S}_i, \boldsymbol{D}) = \mathbb{P}(Y|E_i, \widetilde{\boldsymbol{V}}_i, \boldsymbol{T}\cup\boldsymbol{S}_i) = \mathbb{E}_{\widetilde{X}_i|E_i,\widetilde{\boldsymbol{V}}_i,\boldsymbol{T},\boldsymbol{S}_i}\mathbb{P}(Y|\widetilde{X}_i, E_i, \widetilde{\boldsymbol{V}}_i, \boldsymbol{T}\cup\boldsymbol{S}_i) \\
=\; &\mathbb{P}(Y|\widetilde{X}_i, E_i\cup\boldsymbol{S}_i, \widetilde{\boldsymbol{V}}_i, \boldsymbol{T}\setminus\boldsymbol{S}_i) = \mathbb{P}(Y|\widetilde{X}_i, \widetilde{\boldsymbol{V}}_i, \boldsymbol{T}\setminus\boldsymbol{S}_i) = \mathbb{P}(Y|\widetilde{X}_i, \boldsymbol{D}).
\end{aligned}$$

The third equality follows because $\widetilde{X}_i$ is a constant given E_i and $\text{Pa}(\widetilde{X}_i) = \widetilde{\boldsymbol{V}}_i \cup \boldsymbol{S}_i$. For the fourth equality, all paths between $\boldsymbol{S}_i$ and Y are blocked by $\widetilde{X}_i \cup \widetilde{\boldsymbol{V}}_i \cup \boldsymbol{T}\setminus\boldsymbol{S}_i$ under exchangeability. We thus have $Y \perp\!\!\!\perp_d (E_i\cup\boldsymbol{S}_i)|(\widetilde{X}_i, \widetilde{\boldsymbol{V}}_i, \boldsymbol{T}\setminus\boldsymbol{S}_i)$ and $Y \perp\!\!\!\perp (E_i\cup\boldsymbol{S}_i)|(\widetilde{X}_i, \widetilde{\boldsymbol{V}}_i, \boldsymbol{T}\setminus\boldsymbol{S}_i)$ by the global Markov property. ☐

Lemma 2. *Assume d-separation faithfulness and relevance. Then, (1) $\boldsymbol{T}\cup\widetilde{\boldsymbol{R}}$ contains all of the ancestors of Y in $\boldsymbol{S}\cup\widetilde{\boldsymbol{X}}$, and (2) $(\text{Mb}(\widetilde{R}_i)\cap\widetilde{\boldsymbol{X}}) \subseteq (\widetilde{\boldsymbol{R}}\setminus\widetilde{R}_i)\cup\widetilde{\boldsymbol{N}}$ for any $\widetilde{R}_i \in \widetilde{\boldsymbol{R}}$.*

Proof. We first prove (1). If S_i is an ancestor of Y, then $S_i \not\perp\!\!\!\perp_d Y$, so $S_i \not\perp\!\!\!\perp Y$ by d-separation faithfulness. It follows that $S_i \in \boldsymbol{T}$ by Line 1 of Algorithm 2. If $\widetilde{X}_i$ is an ancestor of Y, then so is $\boldsymbol{S}_i \subseteq \boldsymbol{T}$. Hence $\widetilde{X}_i \not\perp\!\!\!\perp_d \boldsymbol{T}$, so $\widetilde{X}_i \not\perp\!\!\!\perp \boldsymbol{T}$ and $\widetilde{X}_i \in \widetilde{\boldsymbol{R}}$ by d-separation faithfulness and Line 2, respectively. We chose S_i and $\widetilde{X}_i$ arbitrarily, so the set $\boldsymbol{T}\cup\widetilde{\boldsymbol{R}}$ contains all of the ancestors of Y in $\boldsymbol{S}\cup\widetilde{\boldsymbol{X}}$.

We now prove (2). We need to show that $(\widetilde{\boldsymbol{R}}\setminus\widetilde{R}_i)\cup\widetilde{\boldsymbol{N}}$ contains the parents, children and spouses of any $\widetilde{R}_i$, provided that these relatives are also in $\widetilde{\boldsymbol{X}}$. Note that $\widetilde{R}_i \not\perp\!\!\!\perp_d \boldsymbol{T}$ by Line 2 under the global Markov property. Hence, the parents and children of $\widetilde{R}_i$ in $\widetilde{\boldsymbol{X}}$ are also d-connected to $\boldsymbol{T}$ and hence dependent on $\boldsymbol{T}$ under d-separation faithfulness. It follows that $\widetilde{\boldsymbol{R}}\setminus\widetilde{R}_i$ contains all of the parents and children of $\widetilde{R}_i$ also by Line 2. Next, suppose $\widetilde{\boldsymbol{R}}\setminus\widetilde{R}_i$ does not contain a spouse of $\widetilde{R}_i$, which we denote by $\widetilde{X}_j$. Then we have $\widetilde{X}_j \to \widetilde{R}_i \leftarrow S_i$ and $S_i \in \boldsymbol{T}$ under relevance. Hence $X_j \not\perp\!\!\!\perp_d \boldsymbol{T}|\widetilde{\boldsymbol{R}}$, so $X_j \not\perp\!\!\!\perp \boldsymbol{T}|\widetilde{\boldsymbol{R}}$ by d-separation faithfulness and $X_i \in \boldsymbol{N}$ by Line 3. It follows that $\widetilde{\boldsymbol{N}}\cup(\widetilde{\boldsymbol{R}}\setminus\widetilde{R}_i)$ contains all of the spouses of $\widetilde{R}_i$. We conclude that $(\widetilde{\boldsymbol{R}}\setminus\widetilde{R}_i)\cup\widetilde{\boldsymbol{N}}$ contains all members of $\text{Mb}(\widetilde{R}_i)\cap\widetilde{\boldsymbol{X}}$ of any $\widetilde{R}_i \in \widetilde{\boldsymbol{R}}$. ☐

Lemma 8. *Under d-separation faithfulness, relevance and exchangeability, (1)* $T_j \notin \text{Anc}(Q_i)$ *if and only if* $Q_i \perp\!\!\!\perp T_j | \boldsymbol{T} \setminus T_j$ *and (2)* $Q_i \not\perp\!\!\!\perp T_j | (\widetilde{\boldsymbol{Q}} \setminus \widetilde{Q}_i, \widetilde{\boldsymbol{N}}, \boldsymbol{T} \setminus T_j)$ *and* $Q_i \not\perp\!\!\!\perp T_j | \boldsymbol{T} \setminus T_j$ *if and only if* $T_j \in \text{Pa}(\widetilde{Q}_i)$*.*

Proof. For the first statement and forward direction, if $T_j \notin \text{Anc}(Q_i)$, then $Q_i \perp\!\!\!\perp_d T_j | \boldsymbol{T} \setminus T_j$ under exchangeability, so $Q_i \perp\!\!\!\perp T_j | \boldsymbol{T} \setminus T_j$ by the global Markov property. For the backward direction, if $Q_i \perp\!\!\!\perp T_j | \boldsymbol{T} \setminus T_j$, then $Q_i \perp\!\!\!\perp_d T_j | \boldsymbol{T} \setminus T_j$ by d-separation faithfulness. No directed path can thus exist from T_j to Q_i, so $T_j \notin \text{Anc}(Q_i)$.

We next address the second statement. The backward direction follows immediately from d-separation faithfulness. For the forward direction, if $Q_i \not\perp\!\!\!\perp T_j | \boldsymbol{T} \setminus T_j$, then $T_j \in \text{Anc}(Q_i)$ from statement (1). Furthermore, if $Q_i \not\perp\!\!\!\perp T_j | (\widetilde{\boldsymbol{Q}} \setminus \widetilde{Q}_i, \widetilde{\boldsymbol{N}}, \boldsymbol{T} \setminus T_j)$ then $Q_i \not\perp\!\!\!\perp_d T_j | (\widetilde{\boldsymbol{Q}} \setminus \widetilde{Q}_i, \widetilde{\boldsymbol{N}}, \boldsymbol{T} \setminus T_j)$ under the global Markov property. Note that $(\widetilde{\boldsymbol{Q}} \setminus \widetilde{Q}_i) \cup \widetilde{\boldsymbol{N}} \cup \boldsymbol{T}$ contains $\text{Mb}(\widetilde{Q}_i) \cap \widetilde{\boldsymbol{X}}$ by Lemma 2 under d-separation faithfulness and relevance. Therefore, if T_j is not in the Markov boundary of $\widetilde{Q}_i$, then $(\widetilde{\boldsymbol{Q}} \setminus \widetilde{Q}_i) \cup \widetilde{\boldsymbol{N}} \cup \boldsymbol{T} \setminus T_j$ contains $\text{Mb}(\widetilde{Q}_i) \cap (\widetilde{\boldsymbol{X}} \cup \boldsymbol{T})$. As a result, all paths between T_j and $\widetilde{Q}_i$ are blocked by $(\widetilde{\boldsymbol{Q}} \setminus \widetilde{Q}_i) \cup \widetilde{\boldsymbol{N}} \cup \boldsymbol{T} \setminus T_j$ under exchangeability. We thus arrive at the contradiction $Q_i \perp\!\!\!\perp_d T_j | (\widetilde{\boldsymbol{Q}} \setminus \widetilde{Q}_i, \widetilde{\boldsymbol{N}}, \boldsymbol{T} \setminus T_j)$. It follows that T_j must be in the Markov boundary of $\widetilde{Q}_i$ and therefore can only be a parent or a spouse of $\widetilde{Q}_i$ (or both). If T_j is a spouse but not a parent of $\widetilde{Q}_i$, then we arrive at another contradiction that $T_j \notin \text{Anc}(Q_i)$. Hence $T_j \in \text{Pa}(\widetilde{Q}_i)$. □

Lemma 3. *Assume d-separation faithfulness, relevance and exchangeability. Further assume that* $\widetilde{Q}_i$ *is a sink vertex. Then,* $|\Delta_{ij}\gamma_{ij}| \geq \max \Delta^2_{-ij}$ *if and only if* $T_j \notin \text{Anc}(\boldsymbol{Q} \setminus Q_i)$ *or* $T_j \in \text{Pa}(\widetilde{Q}_i)$ *(or both).*

Proof. Assume $|\Delta_{ij}\gamma_{ij}| \geq \max \Delta^2_{-ij}$ for the forward direction. We have two cases. If $|\Delta_{ij}\gamma_{ij}| > 0$, then $Q_i \not\perp\!\!\!\perp T_j | (\widetilde{\boldsymbol{Q}} \setminus \widetilde{Q}_i, \widetilde{\boldsymbol{N}}, \boldsymbol{T} \setminus T_j)$ and $Q_i \not\perp\!\!\!\perp T_j | \boldsymbol{T} \setminus T_j$, so $T_j \in \text{Pa}(\widetilde{Q}_i)$ by Lemma 8. If $|\Delta_{ij}\gamma_{ij}| = 0$, then $\max \Delta^2_{-ij} = 0$, so $Q_k \perp\!\!\!\perp T_j | \boldsymbol{T} \setminus T_j$ for all $Q_k \in \boldsymbol{Q} \setminus Q_i$. We conclude that $T_j \notin \text{Anc}(\boldsymbol{Q} \setminus Q_i)$ by again invoking Lemma 8.

For the backward direction, if $T_j \notin \text{Anc}(\boldsymbol{Q} \setminus Q_i)$, then $Q_k \perp\!\!\!\perp T_j | \boldsymbol{T} \setminus T_j$ for all $Q_k \in \boldsymbol{Q} \setminus Q_i$ by Lemma 8. Thus $\max \Delta^2_{-ij} = 0$ so $|\Delta_{ij}\gamma_{ij}| \geq \max \Delta^2_{-ij}$. If $T_j \in \text{Pa}(\widetilde{Q}_i)$, then $T_j \notin \text{Anc}(\boldsymbol{Q} \setminus Q_i)$ because $\widetilde{Q}_i$ is a sink vertex. Hence $Q_k \perp\!\!\!\perp T_j | \boldsymbol{T} \setminus T_j$ for all $Q_k \in \boldsymbol{Q} \setminus Q_i$ by Lemma 8, so $\max \Delta^2_{-ij} = 0$. We conclude that $|\Delta_{ij}\gamma_{ij}| \geq \max \Delta^2_{-ij}$. □

Lemma 4. $\widetilde{R}_i$ *is a sink vertex if and only if* $R_i \perp\!\!\!\perp (\boldsymbol{T} \setminus \boldsymbol{U}_i) | (\widetilde{\boldsymbol{R}} \setminus \widetilde{R}_i) \cup \widetilde{\boldsymbol{N}} \cup \boldsymbol{U}_i$ *in Line 12 of ACO under d-separation faithfulness, relevance and exchangeability.*

Proof. Assume that $\widetilde{R}_i$ is a sink vertex for the forward direction. We have two cases:

1. If $\widetilde{R}_i \in \text{Anc}(Y)$, then $\text{Pa}(\widetilde{R}_i) \subseteq (\widetilde{\boldsymbol{R}} \setminus \widetilde{R}_i) \cup \boldsymbol{U}_i$ by the first statement of Lemma 2 and Lemma 3. Note that $\widetilde{\boldsymbol{N}}$ Hence, $R_i \perp\!\!\!\perp_d (\boldsymbol{T} \setminus \boldsymbol{U}_i) | (\widetilde{\boldsymbol{R}} \setminus \widetilde{R}_i) \cup \widetilde{\boldsymbol{N}} \cup \boldsymbol{U}_i$ because $\widetilde{R}_i$ is a sink vertex, and $R_i \perp\!\!\!\perp (\boldsymbol{T} \setminus \boldsymbol{U}_i) | (\widetilde{\boldsymbol{R}} \setminus \widetilde{R}_i) \cup \widetilde{\boldsymbol{N}} \cup \boldsymbol{U}_i$ in Line 12 by the global Markov property.
2. If $\widetilde{R}_i \notin \text{Anc}(Y)$, then $(\widetilde{\boldsymbol{R}} \setminus \widetilde{R}_i) \cup \widetilde{\boldsymbol{N}} \cup \boldsymbol{U}_i$ contains all of the parents of $\widetilde{R}_i$ in $\widetilde{\boldsymbol{X}}$ and $\boldsymbol{T}$ by Lemma 2 and Lemma 3, respectively. Moreover, the other direct causal variants of $\widetilde{R}_i$, or $\boldsymbol{S}_i \setminus \boldsymbol{T}$, share no latent confounders with $\boldsymbol{T}$ or any other direct causal variant set excluding $\boldsymbol{T}$ by exchangeability. Hence, we also have $R_i \perp\!\!\!\perp_d (\boldsymbol{T} \setminus \boldsymbol{U}_i) | (\widetilde{\boldsymbol{R}} \setminus \widetilde{R}_i) \cup \widetilde{\boldsymbol{N}} \cup \boldsymbol{U}_i$, and $R_i \perp\!\!\!\perp (\boldsymbol{T} \setminus \boldsymbol{U}_i) | (\widetilde{\boldsymbol{R}} \setminus \widetilde{R}_i) \cup \widetilde{\boldsymbol{N}} \cup \boldsymbol{U}_i$ in Line 12 by the global Markov property.

We have exhausted all possibilities and thus conclude that $R_i \perp\!\!\!\perp (\boldsymbol{T} \setminus \boldsymbol{U}_i) | (\widetilde{\boldsymbol{R}} \setminus \widetilde{R}_i) \cup \widetilde{\boldsymbol{N}} \cup \boldsymbol{U}_i$.

For the backward direction, assume $R_i \perp\!\!\!\perp (\boldsymbol{T} \setminus \boldsymbol{U}_i) | (\widetilde{\boldsymbol{R}} \setminus \widetilde{R}_i) \cup \widetilde{\boldsymbol{N}} \cup \boldsymbol{U}_i$ so that $R_i \perp\!\!\!\perp_d (\boldsymbol{T} \setminus \boldsymbol{U}_i) | (\widetilde{\boldsymbol{R}} \setminus \widetilde{R}_i) \cup \widetilde{\boldsymbol{N}} \cup \boldsymbol{U}_i$ by d-separation faithfulness. Assume for a contradiction that $\widetilde{R}_i$ is not a sink vertex. Then there exists a path $\widetilde{R}_i \to \widetilde{R}_j \leftarrow T_k$ for some $T_k \in \boldsymbol{S}_j \cap \boldsymbol{T}$ by relevance. We thus have $\Delta_{ik} = 0$ but $\Delta^2_{jk} > 0$, so $T_k \notin \boldsymbol{U}_i$ and $T_k \in \boldsymbol{T} \setminus \boldsymbol{U}_i$. We arrive at the contradiction $R_i \not\perp\!\!\!\perp_d (\boldsymbol{T} \setminus \boldsymbol{U}_i) | (\widetilde{\boldsymbol{R}} \setminus \widetilde{R}_i) \cup \widetilde{\boldsymbol{N}} \cup \boldsymbol{U}_i$. The variable $\widetilde{R}_i$ must therefore be a sink vertex. □

Lemma 5. *Under d-separation faithfulness, relevance and exchangeability, ACO recovers the correct causal order* $\boldsymbol{K}$ *over* $\widetilde{\boldsymbol{R}}$ *and* $(\boldsymbol{S}_i \cap \boldsymbol{T}) \subseteq \boldsymbol{P}_i$ *for all* $R_i \in \widetilde{\boldsymbol{R}}$*.*

Proof. We use proof by induction. Base: Suppose $\widetilde{\boldsymbol{R}}$ contains one variable $\widetilde{R}_i$. Then $\boldsymbol{K} = (R_i, Y)$ because R_i is trivially the most independent variable in $\boldsymbol{R}$ according to $\boldsymbol{C}$ of Line 15. The variable R_i is a sink vertex after Y is eliminated, so we have $(\boldsymbol{S}_i \cap \boldsymbol{T}) \subseteq \boldsymbol{P}_i$ under d-separation faithfulness, relevance and exchangeability by Lemma 3. Step: Assume that the conclusion holds when $\widetilde{\boldsymbol{R}}$ contains $p-1$ variables. We need to prove the statement when $\widetilde{\boldsymbol{R}}$ contains p variables. Assume for now that $\widetilde{R}_p$ is an arbitrary sink vertex in $\widetilde{\boldsymbol{R}}$. Lemma 3 then guarantees $|\Delta_{pj}\gamma_{pj}| \geq \max \Delta^2_{-pj}$ for each $S_j \in \boldsymbol{S}_p \cap \boldsymbol{T}$ in Line 8 under d-separation faithfulness, relevance and exchangeability. We thus have $(\boldsymbol{S}_p \cap \boldsymbol{T}) \subseteq \boldsymbol{P}_p$ and no variant of any other parent set is in $\boldsymbol{P}_p$. Finally, the measure of dependence C_p in Line 15 identifies R_p as a sink vertex by Lemma 4. ACO thus eliminates R_p from $\boldsymbol{R}$ and appends it to the front of $\boldsymbol{K}$. The conclusion follows by the inductive hypothesis. □

Lemma 6. *Under d-separation faithfulness, relevance and exchangeability, the graph discovery algorithm outputs the true sub-DAG over $\widetilde{\boldsymbol{R}}$ given a conditional independence oracle, $\boldsymbol{K}$ and $\mathcal{P}$.*

Proof. The set $\widetilde{\boldsymbol{N}} \cup \widetilde{\boldsymbol{R}}$ contains all of the parents of any $\widetilde{R}_i \in \widetilde{\boldsymbol{R}}$ in $\widetilde{\boldsymbol{X}}$ by Lemma 2. Furthermore, $\boldsymbol{P}_i$ contains all of the parents of $\widetilde{R}_i$ in $\boldsymbol{T}$ for any $\widetilde{R}_i \in \widetilde{\boldsymbol{R}}$ by Lemma 4. The stabilized skeleton discovery procedure of the PC algorithm thus recovers all and only the undirected edges in the true DAG over $\widetilde{\boldsymbol{R}}$ under d-separation faithfulness and exchangeability[67]. The conclusion follows because ACO recovers the true causal order over $\widetilde{\boldsymbol{R}}$ also by Lemma 5, so Algorithm 5 infers the true sub-DAG uniquely over $\widetilde{\boldsymbol{R}}$ in Line 19. □

Lemma 7. *Under d-separation faithfulness, relevance and exchangeability, $\widetilde{R}_i$ causes Y – and likewise the vertices $\boldsymbol{S}_i \cup E_i$ cause Y – if and only if $Y \not\perp\!\!\!\perp \widetilde{R}_i | \widetilde{\boldsymbol{V}}_i \cup \boldsymbol{P}_i$.*

Proof. Recall that $(\boldsymbol{S}_i \cap \boldsymbol{T}) \subseteq \boldsymbol{P}_i$ by Lemma 5 under d-separation faithfulness, relevance and exchangeability.

Now if $\widetilde{R}_i$ causes Y, then there exists a directed path from $\widetilde{R}_i$ to Y so $Y \not\perp\!\!\!\perp_d \widetilde{R}_i | \widetilde{\boldsymbol{V}}_i \cup \boldsymbol{P}_i$. We then have $Y \not\perp\!\!\!\perp \widetilde{R}_i | \widetilde{\boldsymbol{V}}_i \cup \boldsymbol{P}_i$ by d-separation faithfulness.

For the backward direction, assume that $\widetilde{R}_i$ does not cause Y. All paths between $\widetilde{R}_i$ and Y are blocked by $\widetilde{\boldsymbol{V}}_i \cup \boldsymbol{P}_i$ under exchangeability. Thus $\widetilde{R}_i$ and Y are d-separated given $\widetilde{\boldsymbol{V}}_i \cup \boldsymbol{P}_i$. We invoke the global Markov property to conclude that $Y \perp\!\!\!\perp \widetilde{R}_i | \widetilde{\boldsymbol{V}}_i \cup \boldsymbol{P}_i$. □

Theorem 1. *(Fisher consistency) Under d-separation faithfulness, relevance and exchangeability, TWRCI identifies all of the direct causal variants of $Y \cup (\mathrm{Anc}(Y) \cap \widetilde{\boldsymbol{X}})$, the unique causal graph over $Y \cup (\mathrm{Anc}(Y) \cap \widetilde{\boldsymbol{X}})$ and the CRCEs of $\mathrm{Anc}(Y) \cap \widetilde{\boldsymbol{X}}$ almost surely as $N \longrightarrow \infty$ with Lipschitz continuous conditional expectations and a conditional independence oracle.*

Proof. Lemma 2 ensures that $\boldsymbol{T} \cup \widetilde{\boldsymbol{R}}$ from Line 1 of Algorithm 1 contains all of the ancestors of Y in $\boldsymbol{S} \cup \widetilde{\boldsymbol{X}}$. Thus $(\mathrm{Anc}(Y) \cap \widetilde{\boldsymbol{X}}) \subseteq \widetilde{\boldsymbol{R}}$ and $(\mathrm{Anc}(Y) \cap \boldsymbol{S}) \subseteq \boldsymbol{T}$. TWRCI identifies $\boldsymbol{S}_Y \subseteq \boldsymbol{P}_Y$ in Line 2 by Lemma 3 under d-separation faithfulness, relevance and exchangeability. The algorithm also identifies $\boldsymbol{S}_i \subseteq \boldsymbol{P}_i$ for each $\widetilde{R}_i \in \mathrm{Anc}(Y)$ under d-separation faithfulness, relevance and exchangeability in Line 3 by invoking Lemma 5. Furthermore, TWRCI recovers the causal order over $\widetilde{\boldsymbol{R}}$ via Lemma 5. TWRCI thus uniquely recovers the sub-DAG over $\widetilde{\boldsymbol{R}}$ in Line 4 by Lemma 6 and then correctly includes Y in the graph by Lemma 7. TWRCI finally identifies the CRCEs of $\mathrm{Anc}(Y) \cap \widetilde{\boldsymbol{X}}$ almost surely in Line 5 given the recovered DAG over $\widetilde{\boldsymbol{R}} \cup Y$ and $\mathcal{P}$ by Lemma 1. □
